# Oseltamivir-Based Organic Salts and Ionic Liquids: Physicochemical Properties, Cytotoxicity, and Neuraminidase Inhibition

**DOI:** 10.3390/pharmaceutics18101340

**Published:** 2026-10-21

**Authors:** Diogo A. Madeira, Vanessa Correia, Luís Santos, Helena Rebelo-de-Andrade, Luís C. Branco

**Affiliations:** 1 LAQV-REQUIMTE, Chemistry Department, NOVA School of Science and Technology (NOVA FCT), 2829-516 Caparica, Portugal; daa.madeira@campus.fct.unl.pt; 2 Antiviral Resistance Lab, Research & Development Unit, Infectious Diseases Department, Instituto Nacional de Saúde Doutor Ricardo Jorge, IP, Av. Padre Cruz, 1649-016 Lisboa, Portugal; vanessa.correia@insa.min-saude.pt (V.C.); luis.santos@insa.min-saude.pt (L.S.); 3 Host-Pathogen Interaction Unit, Research Institute for Medicines (iMed.ULisboa), Faculty of Pharmacy, Universidade de Lisboa, Av. Professor Gama Pinto, 1649-003 Lisboa, Portugal

**Keywords:** oseltamivir, active pharmaceutical ingredient, ionic liquids, organic salts, influenza virus, neuraminidase inhibitor, antiviral resistance

## Abstract

**Background**: Neuraminidase (NA) inhibitors are widely used for the treatment of influenza, but the emergence of resistance and limitations in drug formulation can compromise their therapeutic performance. This study investigated whether counterion exchange can modulate the physicochemical properties, cytotoxicity, and NA-inhibitory activity of oseltamivir (OS) while preserving its covalent structure. **Methods**: Five OS-based ionic formulations, [OS][C_1_SO_3_], [OS][C_3_SO_3_], [OS][C_6_SO_3_], [OS][TolSO_3_], and [OS][CSA], were synthesized and characterized by NMR and ATR-FTIR spectroscopy, with isolated yields ranging from 84 to 88%. Spectroscopic data confirmed the formation of the desired 1:1 salts and showed that the OS ethyl ester remained chemically intact. The formulations were evaluated for aqueous solubility, octanol/water partitioning, cytotoxicity toward MDCK-SIAT1 cells, and NA-inhibitory activity using a MUNANA-based assay against NAs from A(H1N1)pdm09, its H275Y oseltamivir-resistant variant, A(H3N2), and type B influenza viruses. **Results**: All formulations exhibited low cytotoxicity, with CC_50_ values above 1200 µM. All OS ionic formulations showed lower IC_50_ values than oseltamivir phosphate against all four influenza viruses, with improvements ranging from approximately 2.5- to 393-fold. [OS][TolSO_3_] showed the highest potency, reducing the IC_50_ by approximately 336-fold against H275Y virus NA and 393-fold against influenza B virus NA. IC_90_ values showed the same overall potency ranking, although improvements at 50% inhibition were not always proportionally maintained at 90% inhibition. **Conclusions**: Overall, counterion selection substantially influenced both the physicochemical and biological profiles of OS. [OS][TolSO_3_] provided the highest NA-inhibitory potency, whereas [OS][CSA] exhibited the most balanced overall profile, supporting counterion engineering as a potential approach to optimize oseltamivir-based ionic formulations.

## 1. Introduction

Influenza remains a major public health concern. Seasonal epidemics occur every year and can cause high morbidity and mortality, while occasional pandemics can further increase the impact on populations, healthcare systems, and the economy [1,2]. Most healthy adults recover without complications, but severe disease and viral or secondary bacterial pneumonia are more common in older adults, young children, pregnant women, immunocompromised patients, and people with chronic conditions [3]. Vaccination is the main preventive measure, while antiviral treatment remains important for hospitalized and high-risk patients, outbreak control, and pandemic preparedness [1,2].

Influenza viruses are enveloped members of the Orthomyxoviridae family with segmented, negative-sense, single-stranded RNA genomes [4]. Their high genetic variability is driven by antigenic drift, which occurs in both influenza A and B viruses, and by reassortment-mediated antigenic shift in influenza A viruses [5,6]. This continuing evolution can weaken pre-existing immunity, reduce the match between circulating strains and seasonal vaccines, and generate viruses with pandemic potential. The viral surface glycoproteins hemagglutinin (HA) and neuraminidase (NA) have complementary roles in infection. HA binds terminal sialic-acid residues and mediates entry, whereas NA cleaves sialic acids to release progeny virions, prevent viral aggregation, and facilitate movement through respiratory mucus [7,8,9,10]. Productive viral replication depends on a functional balance between HA receptor binding and NA sialidase activity [7,9].

NA is an established antiviral target because its catalytic function is essential and its active site is highly conserved across influenza A and B viruses [9,11]. The enzyme is a tetrameric glycoprotein whose globular head contains catalytic residues involved in substrate recognition and cleavage. Framework residues around this catalytic core help maintain the geometry required for enzymatic activity, but local substitutions can still change inhibitor binding. Structural features around the active site also differ among NA groups. In several group 1 NAs, opening of the 150-loop creates an adjacent 150-cavity, whereas group 2 NAs generally adopt a more closed conformation [12]. The 150-loop is dynamic, however, and can sample different conformations depending on the protein environment [13,14]. This structural plasticity is relevant both to resistance and to the design of next-generation inhibitors.

Approved NA inhibitors include oseltamivir, zanamivir, peramivir, and laninamivir, although their availability and routes of administration vary [15,16]. These compounds mimic key features of the transition state formed during sialic-acid cleavage and competitively occupy the NA catalytic site [16]. Oseltamivir is the most widely used member of this class because it is orally administered and has an established efficacy and safety profile; clinical benefit is greatest when treatment begins early in the course of infection [15,17,18]. Commercial oseltamivir phosphate is an orally bioavailable prodrug that is hydrolyzed mainly by human carboxylesterase 1 to oseltamivir carboxylate, the active NA inhibitor [19,20,21]. Esterification reduces the polarity of the active carboxylate and improves absorption, whereas the phosphate salt provides a water-soluble solid form suitable for administration [20]. The active metabolite has low plasma-protein binding and is eliminated predominantly by the kidneys [20,21,22].

Oseltamivir activity can be compromised by resistance-associated NA substitutions. H275Y is the best-known marker of reduced oseltamivir susceptibility in N1 viruses because it perturbs the local active-site environment and the conformational rearrangements required for efficient inhibitor binding [23,24]. Although resistance mutations may initially impair viral fitness, permissive substitutions can restore replication and transmission [25]. The global spread of H275Y seasonal A(H1N1) viruses before the 2009 pandemic showed that resistant variants can circulate efficiently even without intense antiviral selection [26]. Other substitutions, including E119V, I223R, and N295S, can also reduce susceptibility in a subtype- and background-dependent manner [24,27]. These observations support continued surveillance of antiviral susceptibility and the exploration of approaches that may improve the performance of existing antivirals.

The therapeutic performance of an active pharmaceutical ingredient (API) depends not only on target affinity but also on properties such as solubility, permeability, stability, and solid-state behavior [28]. Salt formation is a widely used way to modify these characteristics without changing the covalent structure of the API [29]. The resulting pharmaceutical profile is determined by the complete ion pair: counterion selection can influence crystal packing, melting behavior, hygroscopicity, dissolution, lipophilicity, stability, and biological interactions [29]. Rational counterion selection can therefore provide a practical route to formulation optimization, but each salt must be evaluated experimentally.

API organic salts and ionic liquids (API-OSILs) extend this approach by using the drug as one of the ionic components [30,31,32]. Ionic liquids are generally defined as salts that melt below 100 °C, while related materials with higher melting points are usually described as organic salts [30,31]. Their properties can be adjusted through counterion selection, and lower crystal-lattice energy may reduce solid-state problems such as polymorphism [31,32]. Depending on the ion pair, these systems can change aqueous solubility, dissolution, permeability, chemical stability, and biological activity [32,33]. In some cases, both ions may contribute to the biological response [34]. However, ionization alone does not ensure better performance or safety. The identity, concentration, structure, and hydrophobicity of both ions still matter [34].

**Figure 1:**
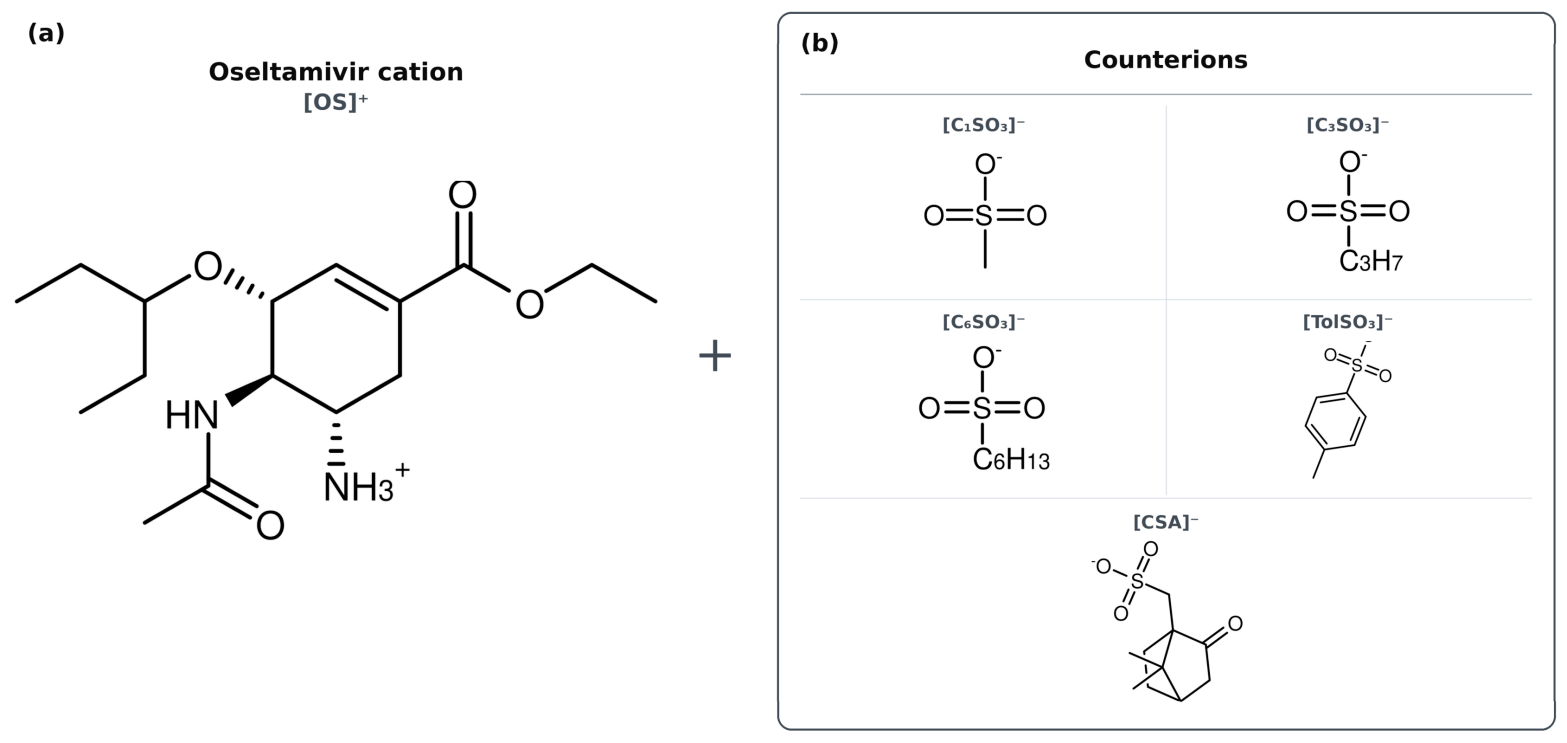
Chemical structures of (**a**) the oseltamivir cation ([OS]^+^) and (**b**) the counterions: methanesulfonate ([C_1_SO_3_]^−^), propanesulfonate ([C_3_SO_3_]^−^), hexanesulfonate ([C_6_SO_3_]^−^), *p*-toluenesulfonate ([TolSO_3_]^−^), and camphorsulfonate ([CSA]^−^).

API-OSIL strategies have been explored particularly for antimicrobial drugs. Ionic formulations of fluoroquinolones, β-lactams, and isoniazid have shown that counterion exchange can modify bioavailability-related properties, cytotoxicity, and antimicrobial activity while retaining the parent API [35,36,37,38,39]. These studies also make clear that greater lipophilicity is not automatically beneficial, because gains in membrane partitioning may be offset by lower aqueous solubility or higher cytotoxicity. Antiviral examples are less common, but ionic forms of acyclovir and hydroxychloroquine have demonstrated improved aqueous solubility or altered in vitro antiviral activity [40,41]. More broadly, ionic formulations have been proposed as a way to revisit existing antiviral APIs whose clinical or formulation performance is constrained by physicochemical properties [42]. This approach has not been systematically applied to oseltamivir, and the effects of organic counterions on its physicochemical properties, cytotoxicity, and NA-inhibitory activity remain poorly defined. In this study, oseltamivir was paired with five sulfonate counterions (Figure 1) differing in alkyl-chain length, hydrophobicity, aromaticity, and structural complexity. The resulting ionic formulations were characterized and evaluated for their physicochemical properties, cytotoxicity, and NA-inhibitory activity against influenza A and B NAs, including the H275Y oseltamivir-resistance substitution. The aim was to determine how counterion selection affects the physicochemical and biological properties of oseltamivir and whether an improved balance between solubility, lipophilicity, cytotoxicity, and NA inhibition could be achieved.

## 2. Materials and Methods

### 2.1. General Remarks

All reagents were purchased from Sigma-Aldrich (St. Louis, MO, USA), Alfa Aesar (Heysham, UK), and TCI (Tokyo, Japan) used as received. The standard solvents were also obtained from Sigma-Aldrich and used without further purification. Amberlyst A-15 resin, with an ion-exchange capacity of 1.1 equiv mL^−1^, was purchased from Supelco (Bellefonte, PA, USA), and deuterium oxide was obtained from Euriso-Top (Saint-Aubin, France). ^1^H and ^13^C NMR spectra were recorded on Bruker AMX400 spectrometers (Bruker, Billerica, MA, USA) at 25 °C using 5 mm borosilicate tubes. Samples were prepared in D_2_O at approximately 5–7 mg mL^−1^. FTIR spectra were recorded in ATR mode over 4000–400 cm^−1^. Chemical shifts are reported in parts per million (ppm). For all compounds, the melting points (mp) were measured using a Stuart Scientific Melting Point SMP1 (Staffordshire, UK). All solvents used for spectroscopic studies were of spectroscopic grade and employed without additional purification. UV–vis absorption spectra. Maximum wavelength was recorded using a Shimadzu UV-3600 spectrophotometer (Kyoto, Japan) with standard 1 cm path length quartz cuvettes.)

### 2.2. General Synthesis of Oseltamivir-Based Organic Salts and Ionic Liquids

Oseltamivir phosphate (300 mg, 0.672 mmol, 1 equiv) was dissolved in 3 mL of saturated aqueous ammonium bicarbonate and stirred for 5 min. The mixture was extracted repeatedly with 8 mL portions of dichloromethane/methanol (3:1, *v*/*v*). The combined organic phases were dried over anhydrous sodium sulfate, filtered, concentrated under reduced pressure, and dried under vacuum to obtain the oseltamivir free base.

The isolated oseltamivir free base (100 mg, 0.244 mmol, 1 equiv) was dissolved in methanol/water (2:1, *v*/*v*). The selected counterion was added in its acid form (1.02 equiv), and the mixture was stirred for 1 h. The solvent was removed under reduced pressure, and the residue was dried under vacuum. For the hexane-1-sulfonate derivative, the corresponding sulfonic acid was generated with Amberlyst A-15 resin before addition to oseltamivir. Residual phosphate was assessed qualitatively by dissolving a small portion of the isolated compound in 3 mL of double-distilled water, acidifying with approximately 1 mL of concentrated nitric acid, and adding ammonium molybdate. A yellow precipitate was interpreted as evidence of residual phosphate; a clear solution indicated that phosphate was absent or below the visual detection limit of the assay [43].

#### 2.2.1. (1S,5R,6R)-6-Acetamido-3-(ethoxycarbonyl)-5-(pentan-3-yloxy)cyclohex-3-en-1-aminium Methanesulfonate, [OS][C_1_SO_3_]

**^1^H NMR** (400 MHz, D_2_O) δ = 6.88 (s, 1H), 4.36 (d, J = 9.1 Hz, 1H), 4.28 (q, J = 6.2 Hz, 2H), 4.08 (m, 1H), 3.72–3.49 (m, 2H), 2.99 (m, 1H), 2.82 (s, 3H), 2.55 (m, 1H), 2.11 (s, 3H), 1.74–1.41 (m, 4H), 1.31 (t, J = 7.1 Hz, 3H), 0.89 (m, 6H).

**^13^C NMR** (101 MHz, D_2_O) δ 175.49, 167.59, 138.33, 127.89, 84.46, 75.35, 62.67, 52.96, 49.45, 28.48, 25.83, 25.40, 22.72, 13.65, 8.94, 8.80.

**FTIR-ATR** (cm^−1^): 3266 (N–H stretch; OS: 3350), 1716 (ester C=O stretch; OS: 1721), 1657 (amide, C=O stretch; OS: 1660), 1166 (asymmetric SO_3_^−^ stretch), and 1040 (symmetric SO_3_^−^ stretch).

**Elemental analysis calcd (%)** for [OS][C_1_SO_3_]·H_2_O: C 48.44, H 7.91, N 6.64; found: C 48.67, H 8.37, N 6.59.

#### 2.2.2. (1S,5R,6R)-6-Acetamido-3-(ethoxycarbonyl)-5-(pentan-3-yloxy)cyclohex-3-en-1-aminium Propane-1-sulfonate, [OS][C_3_SO_3_]

**^1^H NMR** (400 MHz, D_2_O) δ = 6.82 (s, 1H), 4.31–4.19 (m, 3H), 3.92 (m, 1H), 3.53 (m, 1H), 3.47–3.30 (m, 1H), 2.93–2.82 (m, 3H), 2.51–2.32 (m, 1H), 2.07 (s, 3H), 1.90–1.64 (m, 2H), 1.64–1.36 (m, 4H), 1.28 (t, J = 7.1 Hz, 3H), 0.99 (t, J = 7.5 Hz, 3H), 0.85 (m, 6H).

**^13^C NMR** (101 MHz, D_2_O) δ 175.47, 167.57, 138.32, 127.87, 84.44, 75.33, 62.67, 52.95, 49.47, 38.81, 28.48, 25.84, 25.40, 22.72, 13.65, 8.94, 8.81.

**FTIR-ATR** (cm^−1^): 3266 (N–H stretch; OS: 3350), 1718 (ester C=O stretch; OS: 1721), 1656 (amide, C=O stretch; OS: 1660), 1162–1195 (asymmetric SO_3_^−^ stretch), and 1032–1043 (symmetric SO_3_^−^ stretch).

**Elemental analysis calcd (%)** for [OS][C_3_SO_3_]·2H_2_O:C 48.29, H 8.53, N 5.93; found: C 48.97, H 8.36, N 5.87.

#### 2.2.3. (1S,5R,6R)-6-Acetamido-3-(ethoxycarbonyl)-5-(pentan-3-yloxy)cyclohex-3-en-1-aminium Hexane-1-sulfonate, [OS][C_6_SO_3_]

**^1^H NMR** (400 MHz, D_2_O) δ 6.79 (s, 1H), 4.27 (d, J = 9.1 Hz, 1H), 4.18 (m, 2H), 3.98 (t, J = 8.9 Hz, 1H), 3.57–3.43 (m, 2H), 2.89 (m, 1H), 2.82 (m, 2H), 2.56–2.36 (m, 1H), 2.02 (s, 3H), 1.65 (m, 2H), 1.56–1.30 (m, 6H), 1.23 (t, J = 7.1 Hz, 7H), 0.80 (m, 9H).

**^13^C NMR** (101 MHz, D_2_O) δ 175.36, 167.41, 138.40, 127.82, 84.26, 75.26, 62.61, 52.96, 49.47, 31.03, 28.47, 27.88, 25.88, 25.44, 24.41, 22.74, 22.19, 13.71, 13.69, 9.01, 8.83.

**FTIR-ATR** (cm^−1^): 3267 (N–H stretch; OS: 3350), 2961/2934/2875 (aliphatic C–H stretching, consistent with the hexyl chain), 1717 (ester C=O stretch; OS: 1721), 1658 (amide, C=O stretch; OS: 1660), 1194/1162 (asymmetric SO_3_^−^ stretch), and 1036 (symmetric SO_3_^−^ stretch).

**Elemental analysis calcd (%)** for [OS][C_6_SO_3_]·0.5H_2_O: C 54.16, H 9.19, N 5.74; found: C 54.14, H 9.51, N 5.61.

#### 2.2.4. (1S,5R,6R)-6-Acetamido-3-(ethoxycarbonyl)-5-(pentan-3-yloxy)cyclohex-3-en-1-aminium 4-Methylbenzenesulfonate, [OS][TolSO_3_]

**^1^H NMR** (400 MHz, D_2_O) δ 7.70 (d, J = 8.0 Hz, 2H), 7.38 (d, J = 7.9 Hz, 2H), 6.84 (s, 1H), 4.46–4.20 (m, 3H), 4.07 (m, 1H), 3.74–3.40 (m, 2H), 2.96 (m, 1H), 2.67–2.45 (m, 1H), 2.41 (s, 3H), 2.10 (s, 3H), 1.68–1.41 (m, 4H), 1.31 (t, J = 7.1 Hz, 3H), 0.88 (m, 6H).

**^13^C NMR** (101 MHz, D_2_O) δ 175.15, 167.12, 141.87, 141.02, 138.51, 129.56, 127.70, 125.86, 83.89, 75.16, 62.45, 52.96, 49.48, 28.48, 25.94, 25.48, 22.77, 20.86, 13.75, 9.11, 8.85.

**FTIR-ATR** (cm^−1^): 3267 (N–H stretch; OS: 3350), 1717 (ester C=O stretch; OS: 1721), 1657 (amide, C=O stretch; OS: 1660), 1168/1121 (asymmetric SO_3_^−^ stretch), 1033 (symmetric SO_3_^−^ stretch), and 815 (aromatic C–H).

**Elemental analysis calcd (%)** for [OS][TolSO_3_]·H_2_O: C, 55.08; H, 7.45; N, 5.59; found: C 55.06, H 7.52, N 5.54.

#### 2.2.5. (1S,5R,6R)-6-Acetamido-3-(ethoxycarbonyl)-5-(pentan-3-yloxy)cyclohex-3-en-1-aminium ((1S,4R)-7,7-Dimethyl-2-oxobicyclo [2.2.1]heptan-1-yl)methanesulfonate, [OS][CSA]

**^1^H NMR** (400 MHz, D_2_O) δ 6.79 (s, 1H), 4.26 (m, 1H), 4.19 (m, 2H), 3.98 (t, J = 8.9 Hz, 1H), 3.59–3.39 (m, 2H), 3.21 (m, 1H), 2.89 (m, 1H), 2.79 (m, 1H), 2.52–2.26 (m, 3H), 2.09 (t, J = 4.5 Hz, 1H), 2.02 (s, 3H), 2.00–1.84 (m, 1H), 1.68–1.30 (m, 6H), 1.22 (t, J = 7.1 Hz, 3H), 0.97 (s, 3H), 0.87–0.71 (m, 9H).

**^13^C NMR** (101 MHz, D_2_O) δ 175.39, 170.48, 136.89, 129.65, 84.35, 75.54, 58.81, 53.09, 49.73, 47.38, 42.61, 28.97, 26.59, 25.91, 25.49, 24.82, 22.77, 19.28, 19.20, 9.01, 8.90.

**FTIR-ATR** (cm^−1^): 3359 (N–H stretch; OS: 3350), 2967 (aliphatic C–H stretch; OS: 2939, with an additional contribution from the methyl-rich camphor framework), 1745 (camphorsulfonate ketone C=O stretch; absent from OS), 1715 (ester C=O stretch; OS: 1721), 1647 (amide, C=O stretch; OS: 1660), 1554/1526 (amide/N–H deformation region; OS: 1550), 1188/1168 (asymmetric SO_3_^−^ stretch), 1035 (symmetric SO_3_^−^ stretch), and 599/584 (O–S–O deformation).

**Elemental analysis calcd (%)** for [OS][CSA]·3H_2_O: C, 52.16; H, 8.41; N, 4.68; found: C 52.01, H 8.11, N 4.62.

### 2.3. Aqueous Solubility and Octanol/Water Partitioning Assays

Aqueous solubility and octanol/water partitioning were measured by UV-visible spectrophotometry using a calibration curve for each compound, following the general method previously applied to API organic salts [35]. Solubility was measured in water at 37 °C. Saturated samples were equilibrated, undissolved material was removed, and the dissolved concentration was obtained from the corresponding calibration curve.

For the Kow assay, a known concentration of each compound was prepared in water and the initial absorbance (Abi) was recorded. The solution was mixed with an equal volume of 1-octanol and allowed to reach phase equilibrium. After phase separation, the absorbance of the final aqueous phase (Abf) was measured. Under the linear conditions of the assay, the amount transferred to the octanol phase was proportional to Abi − Abf, while the amount remaining in water was proportional to Abf. Kow was calculated as (Abi − Abf)/Abf.

### 2.4. Cytotoxicity Assay

Cytotoxicity was assessed by microscopic examination of the cell monolayer and with measuring cell viability with the CellTiter-Glo^®^ Luminescent Cell Viability Assay (Promega, Madison, WI, USA), following the manufacturer’s instructions.

MDCK-SIAT1 cells (Madin-Darby Canine Kidney cells expressing human β-galactoside α-2,6-sialyltransferase, European Collection of Cell Cultures—ECACC, Cat. N. 05071502, Salisbury, UK), originally described by Matrosovich et al. [44], were seeded in white, flat-bottom 96-well plates at 5 × 10^4^ cells per well in 100 µL and cultured for 24 h to reach 80–90% confluence. Cell treatment was performed with minor modifications to a procedure previously described for hydroxychloroquine API-OSILs [41].

Briefly, OS formulations were prepared as 50 mM stock solutions and serially diluted in infection medium to final concentrations ranging from 1 to 5000 µM. Prior to treatment, cell monolayers were washed twice with prewarmed D-PBS. Each compound dilution (100 µL) was then added in duplicate to the designated wells, while infection medium alone was added to untreated-control and blank (cell-free) wells. Plates were incubated for 72 h at 37 °C in a humidified atmosphere containing 5% CO_2_. To generate a positive cytotoxicity control, 10 µL of Promega lysis solution was added to half of the untreated-control wells 30 min before the addition of the CellTiter-Glo reagent.

At the endpoint, cell morphology was examined under an inverted microscope, and cell viability was quantified with the CellTiter-Glo^®^ assay. Luminescence was measured using a FLUOstar OPTIMA plate reader (BMG Labtech, Madrid, Spain). Raw values were expressed as relative light units (RLUs), and cell viability was calculated as a percentage of the untreated-control. A reduction in cell viability greater than 30% was considered cytotoxic in accordance with ISO 10993–5:2009 [45]. The half-maximal cytotoxic concentration (CC_50_) was determined by point-to-point method in Microsoft Excel. Briefly, the two adjacent experimental concentrations yielding viability values immediately above and below 50% were identified, and the CC_50_ was determined by linear interpolation between these two points of the concentration–response curve. Three independent experiments were performed.

### 2.5. Neuraminidase Inhibition Assays

The NA-inhibition assay was conducted using an established MUNANA-based protocol with minor modifications [46]. The assay was performed against a panel of reference seasonal human influenza viruses. Viral stocks were initially pre-titrated by an NA activity assay, performed as described by Correia et al. (2010) [46], to determine the dilution producing approximately 35,000 RFU (relative fluorescence units) for use in subsequent inhibition assays. This approach minimized inter-strain variability in baseline NA activity and enabled direct comparison of inhibition profiles among influenza viruses.

The inhibition assay was performed on the day of virus pretitration or within the following 24 h. Viral strains were diluted in MES buffer to the predetermined working dilution, and serial dilutions of each OS formulation and its corresponding counterion were prepared in MES buffer over a concentration range of 0.1 to 2000 µM. Compound or counterion dilutions (10 µL) were added in duplicate to the designated wells, followed by 10 µL of the diluted virus. Blank wells received MES buffer in place of virus. Plates were gently agitated and incubated for 30 min at 37 °C with shaking.

Freshly prepared 100 µM MUNANA substrate solution (30 µL) was then added to each well, and the plates were incubated for a further 60 min at 37 °C with shaking in the dark. The reactions were terminated by adding 150 µL of stop solution, and fluorescence was measured using a FLUOstar OPTIMA plate reader. Raw values were expressed as RFUs and plotted against compound concentration in Microsoft Excel. The half-maximal inhibitory concentration (IC_50_), defined as the concentration required to inhibit 50% of NA activity relative to the untreated-virus control, was determined using the point-to-point method by linear interpolation between the two adjacent experimental concentrations yielding inhibition values immediately below and above 50%. Four or five independent experiments were performed, depending on the formulation and influenza virus, to ensure three valid independent values per data point after outlier exclusion, where applicable.

### 2.6. Statistical Analysis

Outliers in CC_50_, IC_50_ and IC_90_ datasets were identified using the scaled median absolute deviation (SMAD). Values deviating from the median by more than 3 × SMAD were classified as outliers and excluded from subsequent analysis. Final CC_50_, IC_50_ and IC_90_ values are reported as arithmetic means of independent assay-level estimates. Graphical representations and statistical analyses were generated in GraphPad Prism version 8.0.2. Differences in CC_50_ and IC_50_ values were evaluated by one-way analysis of variance (ANOVA) followed by Tukey’s multiple-comparisons post hoc test. A *p* value < 0.05 was considered statistically significant.

Fitted IC_90_ and IC_50_ values outside the experimental range were not reported as point estimates: values below 0.1 µM are presented as <0.1 µM and values above 2000 µM as >2000 µM.

## 3. Results and Discussion

### 3.1. Synthesis and Structural Characterization

Phosphate was removed from commercial oseltamivir phosphate under mild conditions to minimize hydrolysis of the ethyl ester. The salt was briefly treated with saturated ammonium bicarbonate and extracted with dichloromethane/methanol. The recovered OS showed the characteristic ethyl ester resonances at approximately 4.12–4.20 and 1.20–1.24 ppm in the ^1^H NMR spectrum, with the expected relative integrals, indicating that the prodrug ester remained intact during phosphate removal. Residual phosphate was assessed qualitatively using an ammonium molybdate test, in which phosphate produces a yellow phosphomolybdate precipitate. No visible precipitate was observed for the isolated OS, indicating that residual phosphate was below the visual detection limit of the assay [43]. Although this test was not quantitative, the result, together with the ^1^H NMR data, supported the use of the isolated material as the OS free base for subsequent salt formation.

The OS free base was subsequently protonated with methanesulfonic acid, propane-1-sulfonic acid, hexane-1-sulfonic acid, 4-methylbenzenesulfonic acid, or camphorsulfonic acid, affording five oseltamivir-based ionic formulations: [OS][C_1_SO_3_], [OS][C_3_SO_3_], [OS][C_6_SO_3_], [OS][TolSO_3_], and [OS][CSA]. All products were isolated as solids in 84–88% yields.

Counterion exchange markedly affected the thermal properties of OS. The resulting formulations exhibited melting points of 76–158 °C, substantially lower than that of commercial OS phosphate (206 °C). [OS][C_3_SO_3_], [OS][C_6_SO_3_], and [OS][TolSO_3_], with melting points below 100 °C, were classified as API ionic liquids, whereas [OS][C_1_SO_3_] and [OS][CSA] were classified as organic salts [30,31,32]. Within the alkylsulfonate series, the melting point decreased progressively with increasing alkyl-chain length, from 158 °C for [OS][C_1_SO_3_] to 97 °C for [OS][C_3_SO_3_] and 76 °C for [OS][C_6_SO_3_]. [OS][TolSO_3_] and [OS][CSA] melted at 80 and 118 °C, respectively. These results highlight the strong influence of counterion identity on intermolecular interactions and crystal packing, despite preservation of the OS covalent structure [30,31,32].

#### Spectroscopic Evidence of OS–Counterion Interaction

The ^1^H NMR spectra confirmed the presence of both OS and the respective counterions in all isolated formulations. Characteristic OS resonances, including the olefinic proton at 6.79–6.88 ppm, the ethyl ester signals at 4.18–4.28 and 1.22–1.31 ppm, and the acetamide methyl group at 2.02–2.11 ppm, were retained in all spectra, confirming preservation of the OS framework and providing no evidence of significant ester hydrolysis. Counterion-specific resonances were also observed, including the methanesulfonate methyl singlet at 2.82 ppm, the aliphatic resonances of the propane- and hexane-sulfonate chains, the aromatic doublets and methyl signal of tosylate, and the characteristic resonances of camphorsulfonate. Their relative integrations were consistent with a 1:1 OS/counterion stoichiometry. Small variations in selected OS chemical shifts, particularly for the olefinic proton and resonances in the 3.9–4.4 ppm region, were observed among the five formulations. As the covalent structure of OS remained unchanged, these differences are consistent with counterion-dependent changes in the local electronic and solvation environments. Given the rapid exchange of ionic species in D_2_O, the ^1^H NMR data support counterion-dependent ionic association but do not establish a unique or persistent ion-pair geometry in solution.

ATR-FTIR spectroscopy provided complementary evidence for salt formation in the solid state. The N–H stretching band shifted from 3350 cm^−1^ in OS phosphate to approximately 3266–3267 cm^−1^ in [OS][C_1_SO_3_], [OS][C_3_SO_3_], [OS][C_6_SO_3_], and [OS][TolSO_3_], indicating a modified hydrogen-bonding environment around the protonated amine. For [OS][C_3_SO_3_], characteristic bands at 3266 cm^−1^ (N–H stretching), 1718 cm^−1^ (ester C=O), and 1656cm^−1^ (amide) were observed, compared with 3350, 1721, and 1660 cm^−1^, respectively, for OS phosphate. The relatively small carbonyl shifts indicate changes in the local environment rather than direct carbonyl–anion interactions. The characteristic SO_3_^−^ stretching bands at 1162–1195 and 1032–1043 cm^−1^ further confirmed incorporation of the sulfonate counterions.

The [OS][CSA] spectrum exhibited a broad N–H stretching band at 3359 cm^−1^ and a characteristic aliphatic C–H stretching band at 2967 cm^−1^. The ketone C=O band of camphorsulfonate at 1745 cm^−1^, absent in OS phosphate, together with the OS ester and amide bands at 1715 and 1647 cm^−1^, respectively, confirmed incorporation of the CSA counterion. Additional bands at 1554 and 1526 cm^−1^, the SO_3_^−^ stretching bands at 1188/1168 and 1035 cm^−1^, and the O–S–O deformation bands at 599/584 cm^−1^ further supported the presence of camphorsulfonate. Likewise, the aromatic C–H band at 815 cm^−1^ served as a characteristic spectral marker for [OS][TolSO_3_].

Overall, the combined ^1^H NMR and ATR-FTIR results consistently support the successful synthesis of the five oseltamivir sulfonate formulations, their approximately 1:1 stoichiometry, and preservation of the oseltamivir ethyl ester. The spectroscopic changes demonstrate that counterion exchange modifies the local chemical environment of OS in the isolated solids while maintaining its covalent structure [30,31,32].

### 3.2. Physicochemical Properties

**Figure 2:**
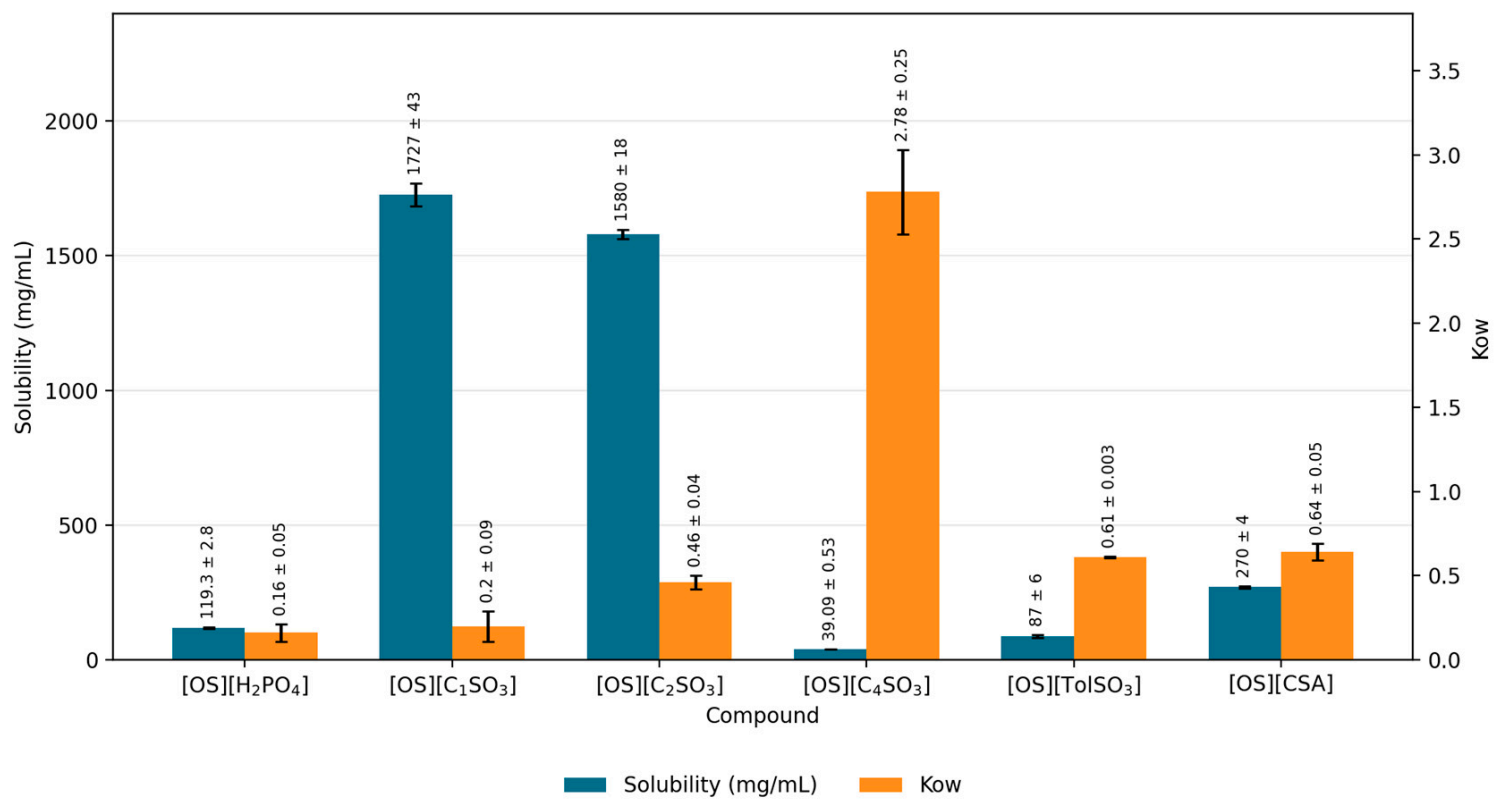
Aqueous solubility and Kow values of oseltamivir phosphate and the five oseltamivir-based ionic formulations. Values are shown as mean ± SD.

Counterion exchange substantially affected both aqueous solubility and octanol/water partitioning of OS (Figure 2). Commercial oseltamivir phosphate showed a Kow of 0.16 ± 0.05 and an aqueous solubility of 119.3 ± 28 mg/mL. [OS][C_1_SO_3_] exhibited a similar Kow (0.20 ± 0.09) but a higher solubility of 1727 ± 43 mg/mL, indicating that methanesulfonate preserved the hydrophilic character of OS while improving its aqueous solubility. [OS][C_3_SO_3_] showed a moderate increase in Kow to 0.46 ± 0.04, approximately 2.9-fold higher than OS phosphate, while maintaining high aqueous solubility (1580 ± 18 mg/mL). Among the alkylsulfonate formulations, [OS][C_3_SO_3_] therefore provided the most favorable balance between increased partitioning into the organic phase and retained aqueous solubility. Similar counterion-dependent effects on the physicochemical properties of API organic salts have been reported previously [30,31,32,33,35,36,37,38,39]. Increasing the alkyl chain length further to six carbon atoms produced a markedly different profile. [OS][C_6_SO_3_] exhibited a Kow of 2.78 ± 0.25, approximately 17-fold higher than OS phosphate, whereas its aqueous solubility decreased sharply to 39.09 ± 0.53 mg/mL. Although the increased Kow indicates greater partitioning into the octanol phase, it should not be interpreted as direct evidence of enhanced membrane permeability. The pronounced reduction in aqueous solubility may, conversely, limit dissolution and consequently affect drug availability. [OS][TolSO_3_] and [OS][CSA] displayed similar Kow values of 0.61 ± 0.003 and 0.64 ± 0.05, respectively, but substantially different aqueous solubilities. [OS][TolSO_3_] showed a solubility of 87 ± 6 mg/mL, whereas [OS][CSA] remained considerably more soluble at 270 ± 4 mg/mL. Thus, formulations with comparable partitioning behavior can exhibit markedly different aqueous solubilities, reflecting the combined effects of hydration, crystal packing, molecular shape, and ion-pair interactions [30,31,32,33,34].

Overall, [OS][C_3_SO_3_] provided the most favorable solubility–lipophilicity balance, combining high aqueous solubility with a moderate increase in Kow. [OS][CSA] also showed a balanced profile, retaining relatively high solubility while moderately increasing partitioning. In contrast, [OS][C_6_SO_3_] and [OS][TolSO_3_] exhibited substantially reduced aqueous solubility. These results demonstrate that the physicochemical behavior of OS-based organic salts cannot be predicted solely from the identity of the counterion. Rather, the observed properties reflect the combined characteristics of the complete ion pair and therefore require experimental assessment for each formulation [30,31,32,33,34]. Importantly, Kow is a descriptor of lipophilicity and should not be considered a direct measure of intestinal permeability. Further evaluation of dissolution, membrane permeability, and ion-pair stability is required before any conclusions regarding oral bioavailability can be drawn [28,29].

### 3.3. Cytotoxicity Assessment

**Figure 3:**
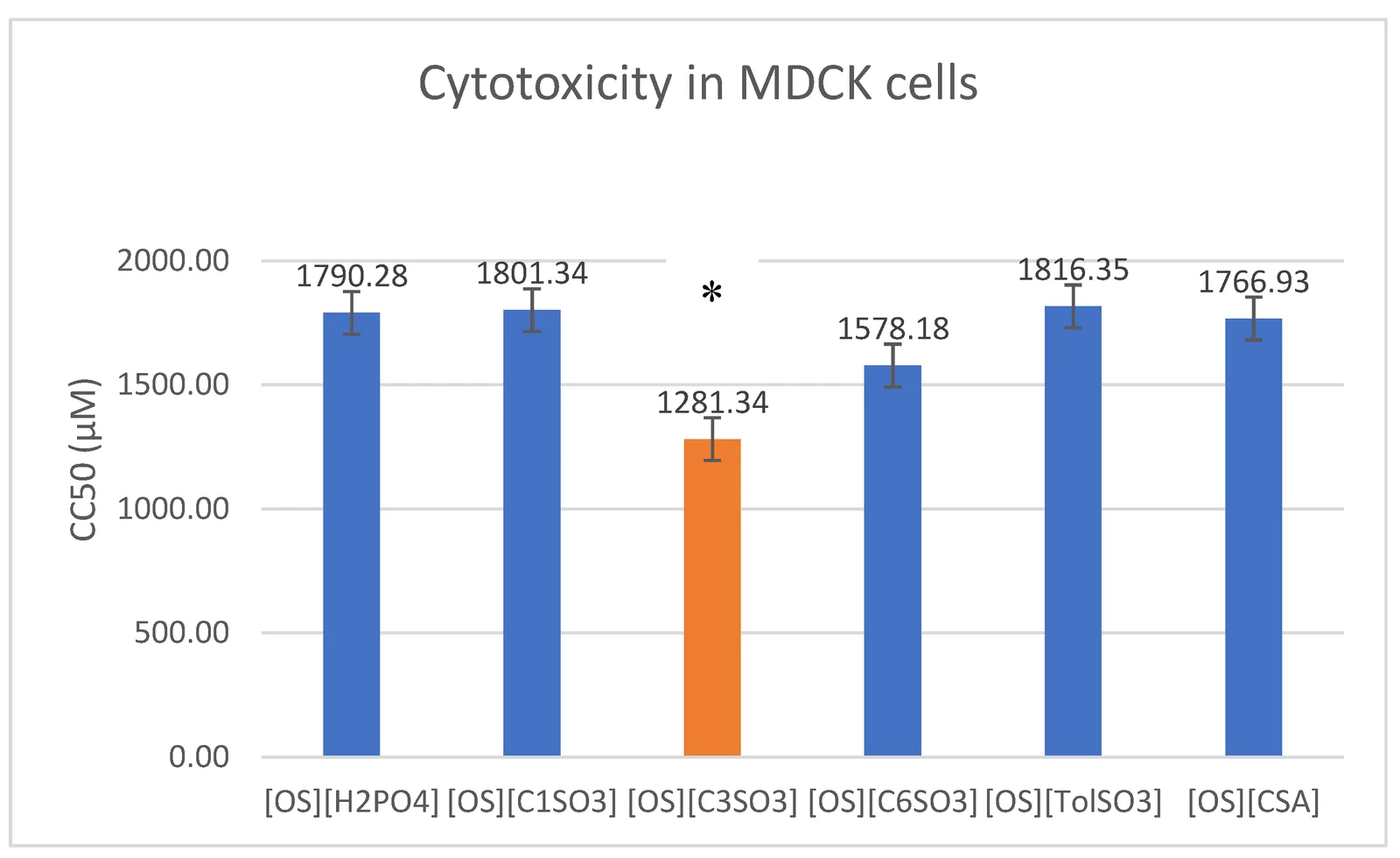
CC_50_ values for oseltamivir phosphate and the five oseltamivir-based formulations in MDCK-SIAT1 cells after 72 h of exposure. Bars show mean ± SD from three independent assays, with individual assay values shown as points. Statistical significance was assessed by one-way ANOVA followed by Tuke’s multiple-comparisons test. * *p* < 0.05 versus oseltamivir phosphate.

OS phosphate exhibited a CC_50_ of 1790.3 µM, consistent with its low cytotoxicity profile reported in previous in vitro studies [47,48]. [OS][C_1_SO_3_], [OS][C_6_SO_3_], [OS][TolSO_3_], and [OS][CSA] displayed CC_50_ values comparable to that of the OS phosphate, ranging from 1578.2 to 1816.3 µM (Figure 3). [OS][C_3_SO_3_] had the lowest CC_50_ (1281.2 µM) and was the only formulation that differed significantly from OS phosphate (*p* < 0.05). Although its CC_50_ value was significantly lower, it remained high, indicating low cytotoxic potential. No clear relationship between formulation lipophilicity and cytotoxicity was observed. Although obtained in a single in vitro cell model, these data provide an important first assessment of the cytotoxicity of the OS-based formulations, which may be further refined in more physiologically relevant systems, particularly human-derived cells.

The counterion salts tested alone showed no detectable cytotoxicity within the tested concentration range. Cell viability remained between 91.3% and 100.9% at 5000 µM, the highest concentration tested, resulting in CC_50_ values > 5000 µM (Figure S19). The low cytotoxicity observed for the counterions supports their suitability for the development of OS-based ionic formulations and is consistent with previous studies using sulfonate and other biocompatible counterions in API organic salts [34,35,37,38,39]. Overall, these results support the preservation of the favorable cytotoxicity profile of OS phosphate across the different ionic formulations.

### 3.4. Neuraminidase Inhibition

**Figure 4:**
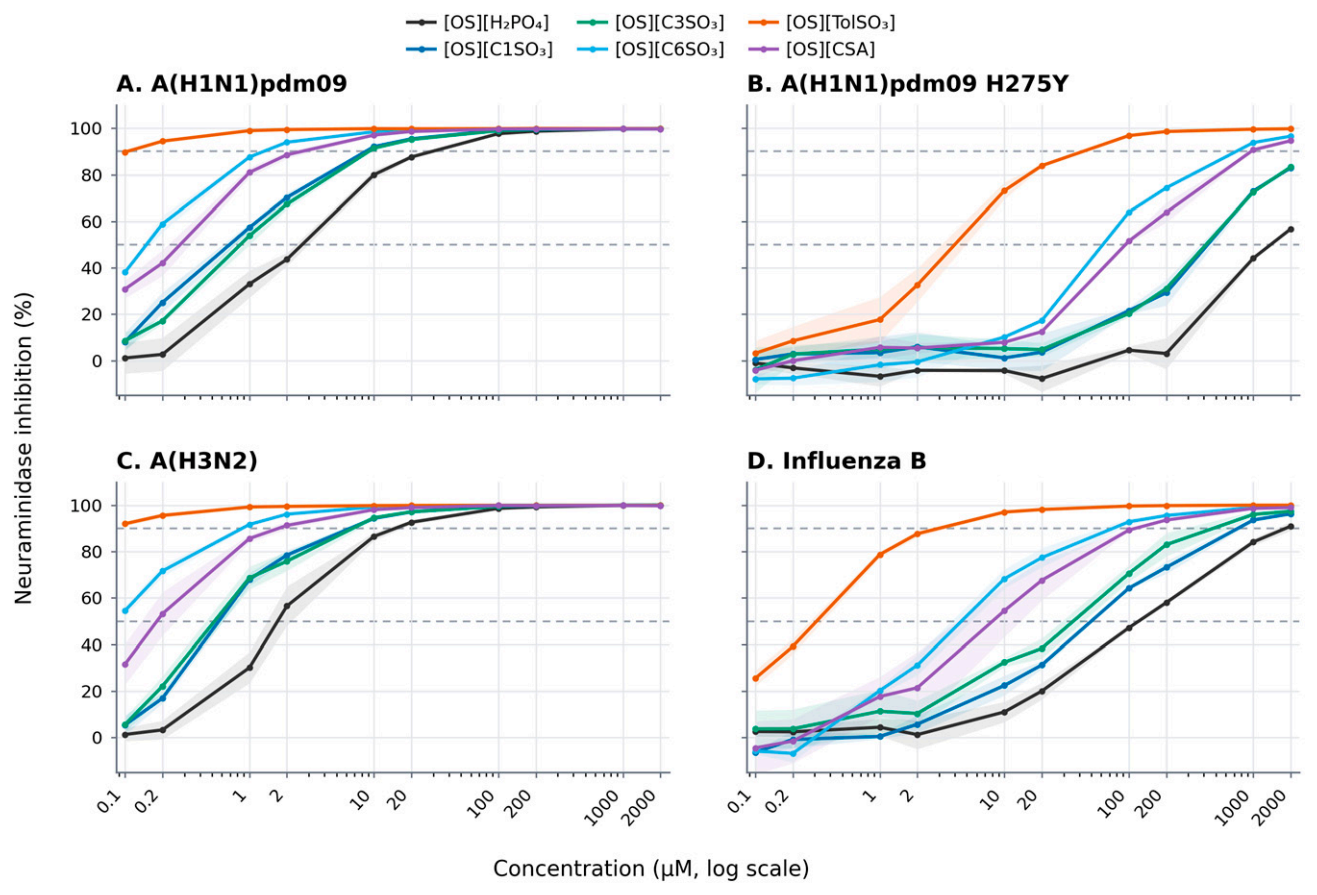
Concentration-dependent neuraminidase inhibition by oseltamivir phosphate and the five oseltamivir-based formulations against (**A**) A(H1N1)pdm09, (**B**) A(H1N1)pdm09 H275Y, (**C**) A(H3N2), and (**D**) influenza B virus. Points show mean inhibition and shaded bands show ± SD across the included independent assays. The horizontal dashed line marks 50% and 90% inhibition.

NA inhibition was evaluated at the 50% and 90% response levels. Mean IC_50_ and IC_90_ values are presented in Table 1 and Table 2, respectively, and the concentration–response curves are shown in Figure 4A–D. While IC_50_ represents the midpoint of the concentration–response curve, IC_90_ is more strongly influenced by curve steepness. Therefore, formulations with similar IC_50_ values did not always require similar concentrations to achieve 90% inhibition.

All OS-OSILs showed lower IC_50_ values than OS phosphate against the four influenza virus NAs tested (Table 1). [OS][TolSO_3_] was the most active formulation, reducing the IC_50_ by >26-fold against A(H1N1)pdm09, 336-fold against its H275Y variant, >19.6-fold against A(H3N2), and 393-fold against influenza B virus, compared to OS phosphate. [OS][C6SO_3_] produced 18.6- to 29.3-fold reductions, followed by [OS][CSA], with improvements ranging from 8.5- to 16-fold. [OS][C_1_SO_3_] and [OS][C_3_SO_3_] showed similar activities, with more moderate 2.4- to 3.7-fold reductions. This overall order, [OS][TolSO_3_] > [OS][C_6_SO_3_] > [OS][CSA] > [OS][C_1_SO_3_] ≈ [OS][C_3_SO_3_], is also reflected by the progressive leftward shift of the curves in Figure 4A–D. When tested individually under the same assay conditions, none of the starting counterion salts showed measurable NA inhibition over the concentration range tested. The increased activity of the OS-OSILs therefore cannot be attributed to an independent inhibitory effect of the counterions alone, but may arise from properties of the formulations.

The OS-OSILs contain a common sulfonate group, but differ markedly in the remaining counterion scaffold, including linear alkyl chains of increasing length (C_1_SO_3_, C_3_SO_3_, and C_6_SO_3_), a non-aromatic bicyclic structure (CSA), and an aromatic ring (TolSO_3_). These structural differences may contribute to the distinct inhibitory potencies observed among the formulations.

Within the linear alkylsulfonate series, the small increase in chain length from one to three carbon atoms ([OS][C_1_SO_3_] and [OS][C_3_SO_3_]) had little effect on NA inhibition, as evidenced by their similar IC_50_ values. However, a further extension to six carbon atoms ([OS][C_6_SO_3_]) resulted in a marked enhancement of inhibitory activity. Given that longer alkyl chains generally increase hydrophobicity while reducing aqueous solubility, these results suggest a positive association between hydrophobicity and activity within this linear sulfonate series. Consistent with this pattern, [OS][CSA] (non-aromatic bicyclic structure) was more active than the C_1_SO_3_- and C_3_SO_3_-based formulations but did not improve upon [OS][C_6_SO_3_]. Although containing ten carbon atoms, CSA possesses a ketone group that increases its polarity and influences its hydration behavior, as evidenced by the lower Kow and higher aqueous solubility of [OS][CSA] compared with [OS][C_6_SO_3_] (see Section 3.2). Consequently, the comparatively lower hydrophobicity of [OS][CSA] may contribute to its lower inhibitory activity. However, hydrophobicity cannot be the sole determinant of NA inhibition. [OS][TolSO_3_] was the most potent formulation despite exhibiting a lower Kow and greater aqueous solubility than [OS][C_6_SO_3_] (Section 3.2), indicating lower overall hydrophobicity. The main structural distinction between the TolSO_3_ and C_6_SO_3_ counterions is the presence of an aromatic ring in TolSO_3_, which provides a more rigid and spatially defined hydrophobic surface than the flexible alkyl chain of C_6_SO_3_. This observation suggests that the inhibitory response of OS-OSILs may depend not only on counterion hydrophobicity but also on its aromatic character, which may contribute to distinct intermolecular interactions with NA. Nevertheless, the NA inhibition assay alone does not provide information on the molecular mechanism underlying the observed enhancement in inhibitory activity. Further structural and computational investigations, including molecular docking studies, will be essential to characterize the interactions between OS-OSILs and influenza virus NA and clarify the molecular basis of the enhanced inhibitory activity.

Some indirect support for this interpretation is provided by previous studies on structurally modified OS derivatives. Compounds [OS][C_3_SO_3_] and [OS][C_6_SO_3_], bearing hydrophobic N-linked substituents, and compound 21h, containing a lipophilic aromatic 4-(phenylthio)benzyl group, displayed enhanced activity relative to OS against influenza A virus NAs, including resistant variants [49,50]. Computational analyses further indicated stronger interactions of these compounds with the NA 150-cavity compared with OS [49]. However, comparison with the present findings should be made cautiously, as these compounds were derived from the active OS carboxylate and contained covalently attached substituents, whereas the OS-OSILs investigated here are based on the ethyl ester prodrug and non-covalently associated sulfonate counterions.

The IC_90_ analysis provided complementary information on the concentrations required to achieve near-complete NA inhibition, confirming the superior inhibitory activity of all five OS-based formulations relative to OS phosphate. Although the improvements observed at IC_50_ were not always maintained to the same extent at the 90% inhibition level, reflecting differences in the slopes of the concentration–response curves, the overall activity trend remained largely consistent. Importantly, the increased inhibitory activity observed at IC_50_ was therefore generally maintained at the higher inhibition threshold, although its magnitude varied among formulations (Table 2).

An apparent selectivity index (SI) was calculated by dividing the CC_50_ values obtained in MDCK-SIAT1 cells by the IC_50_ values determined in the cell-free MUNANA-based NA inhibition assay (Table 3). As these parameters were derived from different experimental systems, the SI values should be considered comparative indicators rather than true selectivity indices.

The SI ranking reflected the trends observed for IC_50_, as expected given the similar CC_50_ values displayed by the formulations. As indicated above (see Section 3.3), [OS][C_3_SO_3_] was the only formulation exhibiting a significantly lower CC_50_ value.

While OSIL formation substantially enhanced NA inhibition, as reflected by lower IC_50_ and IC_90_ values, it did not alter the relative susceptibility of the different virus types/subtypes. Influenza B virus consistently required the highest inhibitor concentrations, followed by A(H1N1)pdm09 and A(H3N2). Although information on the OS ethyl ester prodrug in cell-free NA assays is scarce and limited to A(H1N1) NA (A/PR/8/34) [47], a similar inhibition hierarchy has been widely reported for OS carboxylate, the active metabolite of OS, and is generally attributed to subtype-specific structural differences within the NA enzyme [13,14,51].

The enhanced activity of all OS-OSILs against the OS-resistant A(H1N1)pdm09 H275Y variant is also particularly noteworthy. [OS][TolSO_3_] produced the most substantial effect, yielding a 336-fold reduction in IC_50_ relative to OS phosphate. Although the IC_50_ remained higher than that observed against susceptible A(H1N1)pdm09 virus, this represents a substantial increase in inhibitory activity against resistant variant relative to OS phosphate. Given the continued emergence of OS-resistant influenza strains and the limited availability of effective therapeutic alternatives, these findings support further investigation of OS-OSILs as a strategy of improving the performance of OS-based therapies and addressing emerging antiviral resistance.

The counterion-dependent biological effects observed herein are also consistent with previous findings from our group using hydroxychloroquine-based API-OSIL formulations, which exhibited enhanced in vitro antiviral activity against SARS-CoV-2 [41]. Together, these findings highlight the potential role of counterion engineering in modulating the biological performance of API-OSILs.

Studies are currently underway to investigate the counterions examined herein, along with structurally related analogues of the most promising candidates, in OS carboxylate-based API-OSILs. These studies aim to determine whether the enhanced inhibitory activity observed here for the OS ester formulations can be translated to the pharmacologically active metabolite. Furthermore, they are expected to provide valuable insight into the structure–activity relationships underlying API-counterion interactions and their impact on NA inhibition, physicochemical properties, and cytotoxicity, thereby advancing the rational design of improved antiviral API-OSILs.

## 4. Conclusions

A mild phosphate-removal and protonation strategy enabled the preparation of five OS-based organic salts and ionic liquids in 84–88% yield while preserving the prodrug ethyl ester. ^1^H NMR and ATR-FTIR data confirmed the OS framework, the presence of the intended counterions, and approximately 1:1 salt composition, supporting ionic association in the isolated products without implying a defined ion-pair structure in solution. Counterion exchange substantially modified the thermal, physicochemical, and inhibitory properties of OS phosphate, while preserving its favorable cytotoxicity profile. All five formulations exhibited enhanced NA inhibitory activity compared to OS phosphate, as demonstrated by lower IC_50_ and IC_90_ values against A(H1N1)pdm09, the H275Y OS-resistant variant, A(H3N2), and influenza B viruses. Among them, [OS][TolSO_3_] displayed the greatest improvement in inhibitory potency across the virus panel, whereas [OS][CSA] provided the most balanced overall profile, combining enhanced NA inhibition with favorable physicochemical and cytotoxicity characteristics. Importantly, these results show that counterion selection can provide distinct advantages, allowing optimization towards either greater NA inhibitory potency or a more balanced profile across the properties evaluated. Taken together, these findings demonstrate that counterion engineering can substantially improve the properties and NA-inhibitory activity of OS without modifying its covalent structure, highlighting API-OSIL formation as a promising strategy for optimizing existing antivirals. Complementary structural and computational studies will be essential to characterize how the different counterions influence the molecular interactions of OS with influenza virus NA and to clarify the molecular basis of the observed enhancement in inhibitory activity. Future work should focus on confirming the antiviral activity and biopharmaceutical advantages of these OS-OSILs in relevant cellular models, with the aim of advancing their translational potential.

## Figures and Tables

**Table 1:** IC_50_ values for inhibition of influenza neuraminidase by oseltamivir phosphate and the oseltamivir-based formulations. Values are expressed as arithmetic mean ± SD in µM. * *p* < 0.05 versus OS phosphate.

Formulation	H1N1 (pdm09)	H1N1 (H275Y)	H3N2	B
[OS][H_2_PO_4_]	2.65 ± 0.29	1344 ± 60	2.14 ± 0.52	123.0 ± 2.6
[OS][C_1_SO_3_]	0.710 * ± 0.034	427.1 * ± 47.3	0.595 * ± 0.065	49.88 * ± 1.31
[OS][C_3_SO_3_]	0.718 * ± 0.056	416.2 * ± 8.6	0.540 * ± 0.086	33.60 * ± 3.62
[OS][C_6_SO_3_]	0.140 * ± 0.014	61.79 * ± 0.28	<0.1 *	4.21 * ± 0.70
[OS][TolSO_3_]	<0.1 *	4.00 * ± 0.83	<0.1 *	0.313 * ± 0.038
[OS][CSA]	0.275 * ± 0.044	117.1 * ± 4.8	0.228 * ± 0.065	7.68 * ± 1.49

**Table 2:** IC_90_ values for inhibition of influenza neuraminidase by oseltamivir phosphate and the oseltamivir-based formulations. Values are expressed as arithmetic mean ± SD in µM. Results outside the 0.1–2000 µM test range are shown only as experimental bounds.

Formulation	H1N1 (pdm09)	H1N1 (H275Y)	H3N2	B
[OS][H_2_PO_4_]	26.56 ± 5.84	>2000	15.59 ± 0.68	1654 ± 118
[OS][C_1_SO_3_]	8.58 ± 0.35	>2000	6.41 ± 0.71	700.7 ± 97.1
[OS][C_3_SO_3_]	9.07 ± 0.52	>2000	7.18 ± 0.15	462.2 ± 187.3
[OS][C_6_SO_3_]	1.30 ± 0.07	713.3 ± 2.3	0.869 ± 0.065	73.49 ± 15.01
[OS][TolSO_3_]	0.104 ± 0.005	41.04 ± 0.53	<0.1	2.969 ± 0.581
[OS][CSA]	2.22 ± 0.13	957.5 ± 22.3	1.75 ± 0.36	112.2 ± 7.6

**Table 3:** Apparent selectivity indices calculated from cytotoxicity and neuraminidase-inhibition measurements.

Formulation	Apparent SI (CC_50_/IC_50_)			
	A(H1N1)pdm09	A(H1N1)pdm09 H275Y	A(H3N2)	Influenza B
[OS][H_2_PO_4_]	636.0	1.4	836.6	14.6
[OS][C_1_SO_3_]	2513.5	4.2	3027.5	38.0
[OS][C_3_SO_3_]	1747.3	3.1	2372.8	38.1
[OS][C_6_SO_3_]	11,010.6	25.5	10,884.0	375.3
[OS][TolSO_3_]	>18,163.5	454.5	>18,163.5	5796.9
[OS][CSA]	6425.2	14.0	7766.7	292.0

Apparent SI was calculated as mean CC_50_/mean IC_50_. When IC_50_ was below the 0.1 µM lower test limit, SI was reported as a lower bound calculated with 0.1 µM; therefore, [OS][TolSO_3_] values against A(H1N1)pdm09 and A(H3N2) are >18,163.5. Higher ratios indicate a wider separation between cytotoxicity and NA inhibition. These values are comparative screening indices rather than conventional antiviral selectivity indices because CC_50_ was measured in MDCK-SIAT1 cells, whereas IC_50_ was obtained with the cell-free MUNANA assay.

## Data Availability

The original contributions presented in this study are included in the article/Supplementary Materials. Further inquiries can be directed to the corresponding authors.

## Supplementary Materials

The following supporting information can be downloaded at: https://www.mdpi.com/article/10.3390/pharmaceutics18101340/s1, Figure S1. FTIR-ATR spectrum of [OS][H_2_PO_4_]; Figure S2. FTIR-ATR spectrum of [OS][C_1_SO_3_]; Figure S3. FTIR-ATR spectrum of [OS][C_3_SO_3_]; Figure S4. FTIR-ATR spectrum of [OS][C_6_SO_3_]; Figure S5. FTIR-ATR spectrum of [OS][TolSO_3_]; Figure S6. FTIR-ATR spectrum of [OS][CSA]; Figure S7. 1H NMR spectrum of [OS][H_2_PO_4_] in D_2_O; Figure S8. 1H NMR spectrum of [OS][C_1_SO_3_] in D_2_O; Figure S9. 1H NMR spectrum of [OS][C_3_SO_3_] in D_2_O; Figure S10. 1H NMR spectrum of [OS][C_6_SO_3_] in D_2_O; Figure S11. 1H NMR spectrum of [OS][TolSO_3_] in D_2_O; Figure S12. 1H NMR spectrum of [OS][CSA] in D_2_O; Figure S13. 13C APT NMR spectrum of [OS][H_2_PO_4_] in D_2_O (DMSO-d6 reference); Figure S14. 13C APT NMR spectrum of [OS][C_1_SO_3_] in D_2_O (acetone-d6 reference); Figure S15. 13C APT NMR spectrum of [OS][C_3_SO_3_] in D_2_O (acetone-d6 reference); Figure S16. 13C APT NMR spectrum of [OS][C_6_SO_3_] in D_2_O (acetone-d6 reference); Figure S17. 13C APT NMR spectrum of [OS][TolSO_3_] in D_2_O (acetone-d6 reference); Figure S18. 13C APT NMR spectrum of [OS][CSA] in D_2_O (acetone-d6 reference); Figure S19. Cytotoxicity of the starting counterions (IL1: [K][C_1_SO_3_]; IL2: [Na][C_3_SO_3_]; IL3: [Na][C_6_SO_3_]; IL4: [Na][TolSO_3_]; IL5: [Na][CSA]) in MDCK-SIAT1 cells; Figure S20. UV-Vis absorbance spectra recorded before (Abi) and after (Abf) octanol-water partitioning for [OS][H_2_PO_4_]; Figure S21. UV-Vis absorbance spectra recorded before (Abi) and after (Abf) octanol-water partitioning for [OS][C_1_SO_3_]; Figure S22. UV-Vis absorbance spectra recorded before (Abi) and after (Abf) octanol-water partitioning for [OS][C_3_SO_3_]; Figure S23. UV-Vis absorbance spectra recorded before (Abi) and after (Abf) octanol-water partitioning for [OS][C_6_SO_3_]; Figure S24. UV-Vis absorbance spectra recorded before (Abi) and after (Abf) octanol-water partitioning for [OS][TolSO_3_]; Figure S25. UV-Vis absorbance spectra recorded before (Abi) and after (Abf) octanol-water partitioning for [OS][CSA].

## Abbreviations

ANOVA, analysis of variance; API, active pharmaceutical ingredient; API-OSILs, active pharmaceutical ingredient organic salts and ionic liquids; ATR, attenuated total reflectance; C_1_SO_3_, methanesulfonate; C_3_SO_3_, propane-1-sulfonate; C_6_SO_3_, hexane-1-sulfonate; CC_50_, half-maximal cytotoxic concentration; CSA, camphorsulfonate; D-PBS, Dulbecco’s phosphate-buffered saline; FTIR, Fourier-transform infrared spectroscopy; HA, hemagglutinin; IC_50_, half-maximal inhibitory concentration; IC_90_, 90% inhibitory concentration; ISO, International Organization for Standardization; Kow, octanol–water partition coefficient; MDCK-SIAT1, Madin-Darby canine kidney cells overexpressing human α2,6-sialyltransferase; MES, 2-(N-morpholino)ethanesulfonic acid; MUNANA, 2′-(4-methylumbelliferyl)-α-D-N-acetylneuraminic acid; NA, neuraminidase; NMR, nuclear magnetic resonance; OS, oseltamivir; OS-OSILs, oseltamivir-based organic salts and ionic liquids; OSILs, organic salts and ionic liquids; RFU, relative fluorescence units; RNA, ribonucleic acid; SD, standard deviation; SI, selectivity index; SMAD, scaled median absolute deviation; TolSO_3_, 4-methylbenzenesulfonate; UV, ultraviolet.

## References

[1] World Health Organization Influenza (Seasonal). https://www.who.int/health-topics/influenza-seasonal.

[2] Krammer F., Smith G.J.D., Fouchier R.A.M., Peiris M., Kedzierska K., Doherty P.C., Palese P., Shaw M.L., Treanor J., Webster R.G. (2018). Influenza. Nat. Rev. Dis. Primers.

[3] Rothberg M.B., Haessler S.D., Brown R.B. (2008). Complications of viral influenza. Am. J. Med..

[4] Lamb R.A., Krug R.M., Knipe D.M., Howley P.M. (2013). Orthomyxoviridae: The viruses and their replication. Fields Virology.

[5] Kim H., Webster R.G., Webby R.J. (2018). Influenza virus: Dealing with a drifting and shifting pathogen. Viral Immunol..

[6] Lowen A.C. (2017). Constraints, drivers, and implications of influenza A virus reassortment. Annu. Rev. Virol..

[7] Gamblin S.J., Skehel J.J. (2010). Influenza hemagglutinin and neuraminidase membrane glycoproteins. J. Biol. Chem..

[8] Rogers G.N., Paulson J.C. (1983). Receptor determinants of human and animal influenza virus isolates: Differences in receptor specificity of the H3 hemagglutinin based on species of origin. Virology.

[9] McAuley J.L., Gilbertson B.P., Trifkovic S., Brown L.E., McKimm-Breschkin J.L. (2019). Influenza virus neuraminidase structure and functions. Front. Microbiol..

[10] Cohen M., Zhang X.-Q., Senaati H.P., Chen H.-W., Varki N.M., Schooley R.T., Gagneux P. (2013). Influenza A penetrates host mucus by cleaving sialic acids with neuraminidase. Virol. J..

[11] Colman P.M., Varghese J.N., Laver W.G. (1983). Structure of the catalytic and antigenic sites in influenza virus neuraminidase. Nature.

[12] Russell R.J., Haire L.F., Stevens D.J., Collins P.J., Lin Y.P., Blackburn G.M., Hay A.J., Gamblin S.J., Skehel J.J. (2006). The structure of H5N1 avian influenza neuraminidase suggests new opportunities for drug design. Nature.

[13] Li Q., Qi J., Zhang W., Vavricka C.J., Shi Y., Wei J., Feng E., Shen J., Chen J., Liu D. (2010). The 2009 pandemic H1N1 neuraminidase N1 lacks the 150-cavity in its active site. Nat. Struct. Mol. Biol..

[14] Amaro R.E., Swift R.V., Votapka L., Li W.W., Walker R.C., Bush R.M. (2011). Mechanism of 150-cavity formation in influenza neuraminidase. Nat. Commun..

[15] Kamali A., Holodniy M. (2013). Influenza treatment and prophylaxis with neuraminidase inhibitors: A review. Infect. Drug Resist..

[16] Moscona A. (2005). Neuraminidase inhibitors for influenza. N. Engl. J. Med..

[17] Treanor J.J., Hayden F.G., Vrooman P.S., Barbarash R., Bettis R., Riff D., Singh S., Kinnersley N., Ward P., Mills R.G. (2000). Efficacy and safety of the oral neuraminidase inhibitor oseltamivir in treating acute influenza: A randomized controlled trial. JAMA.

[18] Dobson J., Whitley R.J., Pocock S., Monto A.S. (2015). Oseltamivir treatment for influenza in adults: A meta-analysis of randomised controlled trials. Lancet.

[19] Kim C.U., Lew W., Williams M.A., Liu H., Zhang L., Swaminathan S., Bischofberger N., Chen M.S., Mendel D.B., Tai C.Y. (1997). Influenza neuraminidase inhibitors possessing a novel hydrophobic interaction in the enzyme active site: Design, synthesis, and structural analysis of carbocyclic sialic acid analogues with potent anti-influenza activity. J. Am. Chem. Soc..

[20] He G., Massarella J., Ward P. (1999). Clinical pharmacokinetics of the prodrug oseltamivir and its active metabolite Ro 64-0802. Clin. Pharmacokinet..

[21] Davies B.E. (2010). Pharmacokinetics of oseltamivir: An oral antiviral for the treatment and prophylaxis of influenza in diverse populations. J. Antimicrob. Chemother..

[22] Shi D., Yang J., Yang D., LeCluyse E.L., Black C., You L., Akhlaghi F., Yan B. (2006). Anti-influenza prodrug oseltamivir is activated by carboxylesterase human carboxylesterase 1, and the activation is inhibited by antiplatelet agent clopidogrel. J. Pharmacol. Exp. Ther..

[23] Collins P.J., Haire L.F., Lin Y.P., Liu J., Russell R.J., Walker P.A., Skehel J.J., Martin S.R., Hay A.J., Gamblin S.J. (2008). Crystal structures of oseltamivir-resistant influenza virus neuraminidase mutants. Nature.

[24] Samson M., Pizzorno A., Abed Y., Boivin G. (2013). Influenza virus resistance to neuraminidase inhibitors. Antivir. Res..

[25] Bloom J.D., Gong L.I., Baltimore D. (2010). Permissive secondary mutations enable oseltamivir resistance. Science.

[26] Moscona A. (2009). Global transmission of oseltamivir-resistant influenza. N. Engl. J. Med..

[27] Lackenby A., Besselaar T.G., Daniels R.S., Fry A., Gregory V., Gubareva L.V., Huang W., Hurt A.C., Leang S.-K., Lee R.T.C. (2018). Global update on the susceptibility of human influenza viruses to neuraminidase inhibitors and status of novel antivirals, 2016–2017. Antivir. Res..

[28] Amidon G.L., Lennernäs H., Shah V.P., Crison J.R. (1995). A theoretical basis for a biopharmaceutic drug classification: The correlation of in vitro drug product dissolution and in vivo bioavailability. Pharm. Res..

[29] Serajuddin A.T.M. (2007). Salt formation to improve drug solubility. Adv. Drug Deliv. Rev..

[30] Marrucho I.M., Branco L.C., Rebelo L.P.N. (2014). Ionic liquids in pharmaceutical applications. Annu. Rev. Chem. Biomol. Eng..

[31] Stoimenovski J., MacFarlane D.R., Bica K., Rogers R.D. (2010). Crystalline vs. ionic liquid salt forms of active pharmaceutical ingredients: A position paper. Pharm. Res..

[32] Shamshina J.L., Rogers R.D. (2023). Ionic liquids: New forms of active pharmaceutical ingredients with unique, tunable properties. Chem. Rev..

[33] Handa M., Almalki W.H., Shukla R., Afzal O., Altamimi A.S.A., Beg S., Rahman M. (2022). Active pharmaceutical ingredients (APIs) in ionic liquids: An effective approach for API physiochemical parameter optimization. Drug Discov. Today.

[34] Egorova K.S., Ananikov V.P. (2017). Biological activity of ionic liquids: Applications in pharmaceutics and medicine. Chem. Rev..

[35] Florindo C., Costa A., Matos C., Nunes S.L., Matias A.N., Duarte C.M.M., Rebelo L.P.N., Branco L.C., Marrucho I.M. (2014). Novel organic salts based on fluoroquinolone drugs: Synthesis, bioavailability and toxicological profiles. Int. J. Pharm..

[36] Ferraz R., Teixeira V., Rodrigues D., Fernandes R., Prudêncio C., Noronha J.P., Petrovski Ž., Branco L.C. (2014). Antibacterial activity of ionic liquids based on ampicillin against resistant bacteria. RSC Adv..

[37] Santos F., Branco L.C., Duarte A.R.C. (2020). Organic salts based on isoniazid drug: Synthesis, bioavailability and cytotoxicity studies. Pharmaceutics.

[38] Santos M.M., Alves C., Silva J., Florindo C., Costa A., Petrovski Ž., Marrucho I.M., Pedrosa R., Branco L.C. (2020). Antimicrobial activities of highly bioavailable organic salts and ionic liquids from fluoroquinolones. Pharmaceutics.

[39] Madeira D., Alves C., Silva J., Florindo C., Costa A., Petrovski Ž., Marrucho I.M., Pedrosa R., Santos M.M., Branco L.C. (2021). Fluoroquinolone-based organic salts and ionic liquids as highly bioavailable broad-spectrum antimicrobials. Proceedings.

[40] Shamshina J.L., Kelley S.P., Gurau G., Rogers R.D. (2017). Acyclovir as an ionic liquid cation or anion can improve aqueous solubility. ACS Omega.

[41] Faísca F., Correia V., Petrovski Ž., Branco L.C., Rebelo-de-Andrade H., Santos M.M. (2022). Enhanced in vitro antiviral activity of hydroxychloroquine ionic liquids against SARS-CoV-2. Pharmaceutics.

[42] Shamshina J.L., Rogers R.D. (2020). Are myths and preconceptions preventing us from applying ionic liquid forms of antiviral medicines to the current health crisis?. Int. J. Mol. Sci..

[43] Svehla G. (1996). Vogel’s Qualitative Inorganic Analysis.

[44] Matrosovich M., Matrosovich T., Carr J., Roberts N.A., Klenk H.-D. (2003). Overexpression of the alpha-2,6-sialyltransferase in MDCK cells increases influenza virus sensitivity to neuraminidase inhibitors. J. Virol..

[45] (2009). Biological Evaluation of Medical Devices—Part 5: Tests for In Vitro Cytotoxicity.

[46] Correia V., de Andrade H.R., Santos L.A., Lackenby A., Zambon M. (2010). Antiviral drug profile of seasonal influenza viruses circulating in Portugal from 2004/2005 to 2008/2009 winter seasons. Antivir. Res..

[47] Shin J.S., Ku K.B., Jang Y., Yoon Y.-S., Shin D., Kwon O.S., Go Y.Y., Kim S.S., Bae M.A., Kim M. (2017). Comparison of anti-influenza virus activity and pharmacokinetics of oseltamivir free base and oseltamivir phosphate. J. Microbiol..

[48] Noh M., Cho S.-Y., Choi J., Song S.-H., Cho J.-Y., Vaidya B., Kim D. (2025). Enhanced anti-influenza activity of fermented yellow soybean extract and daidzein co-treatment on MDCK cells. Food Sci. Biotechnol..

[49] Zhang J., Poongavanam V., Kang D., Bertagnin C., Lu H., Kong X., Ju H., Lu X., Gao P., Tian Y. (2018). Optimization of N-Substituted Oseltamivir Derivatives as Potent Inhibitors of Group-1 and -2 Influenza A Neuraminidases, Including a Drug-Resistant Variant. J. Med. Chem..

[50] Zhang J., Murugan N.A., Tian Y., Bertagnin C., Fang Z., Kang D., Kong X., Jia H., Sun Z., Jia R. (2018). Structure-Based Optimization of N-Substituted Oseltamivir Derivatives as Potent Anti-Influenza A Virus Agents with Significantly Improved Potency against Oseltamivir-Resistant N1-H274Y Variant. J. Med. Chem..

[51] Oakley A.J., Barrett S., Peat T.S., Newman J., Streltsov V.A., Waddington L., Saito T., Tashiro M., McKimm-Breschkin J.L. (2010). Structural and Functional Basis of Resistance to Neuraminidase Inhibitors of Influenza B Viruses. J. Med. Chem..

