# Supplementary material for: Oseltamivir-Based Organic Salts and Ionic Liquids: Physicochemical Properties, Cytotoxicity, and Neuraminidase Inhibition
Source: Pharmaceutics. 2026 Oct 21;18(10):1340. doi: 10.3390/pharmaceutics18101340 (PMC13708149; doi:10.3390/pharmaceutics18101340)
Supplement: Supplementary file 1 [file pharmaceutics-18-01340-s001.zip › pharmaceutics-4569932-supplementary.pdf]

Supplementary Materials

FTIR spectra

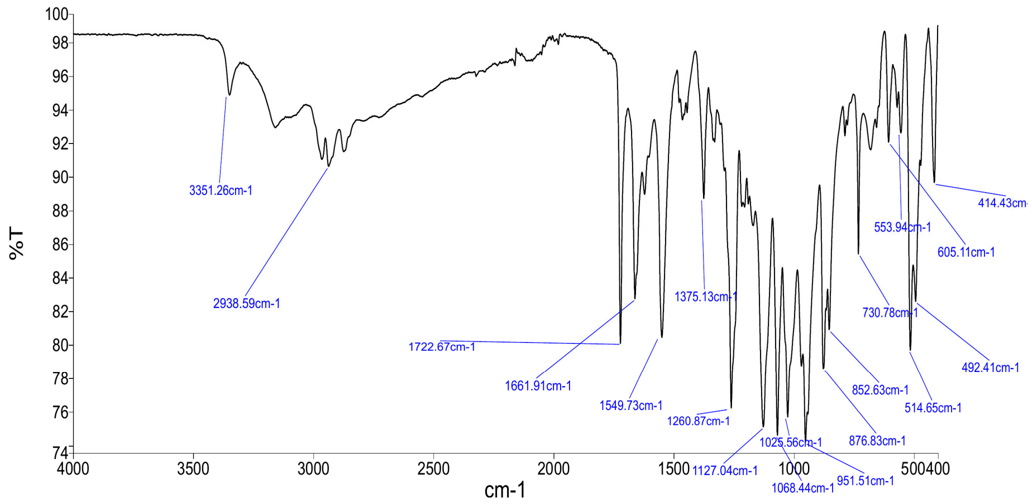

Figure S1. FTIR-ATR spectrum of $[OS][H_2PO_4]$

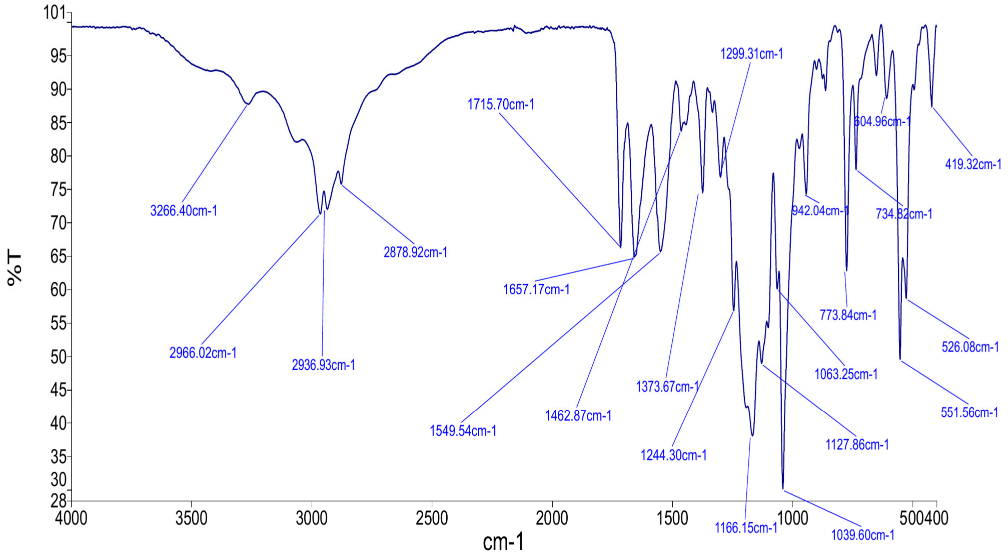

Figure S2. FTIR-ATR spectrum of $[OS][C_1SO_3]$.

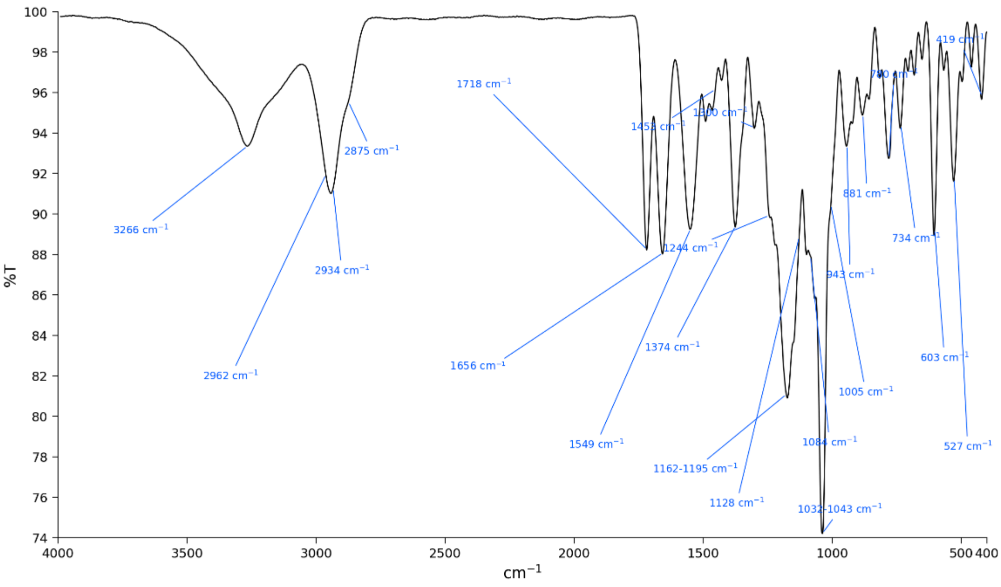

Figure S3. FTIR-ATR spectrum of [OS][C_3SO_3]

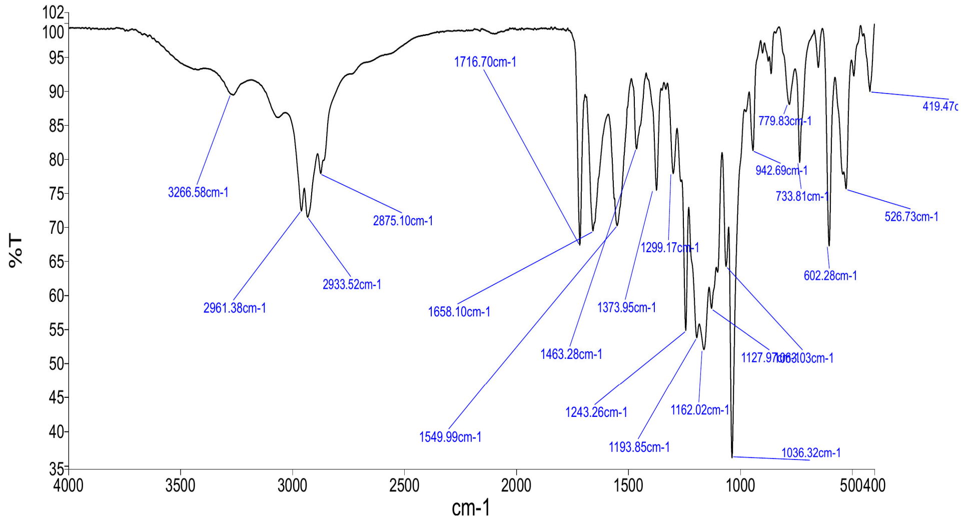

Figure S4. FTIR-ATR spectrum of [OS][C_6SO_3].

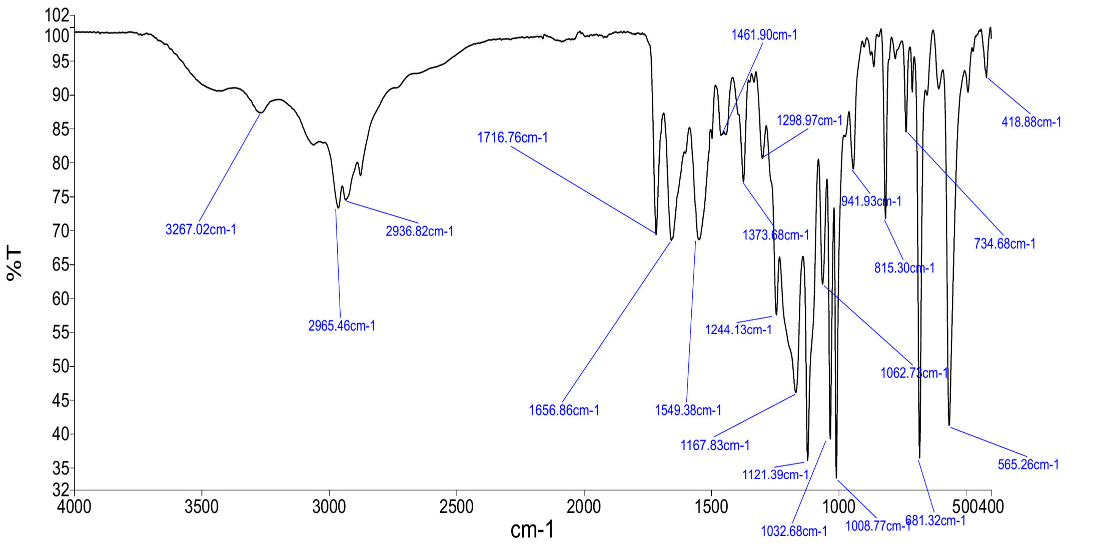

Figure S5. FTIR-ATR spectrum of [OS][TolSO3].

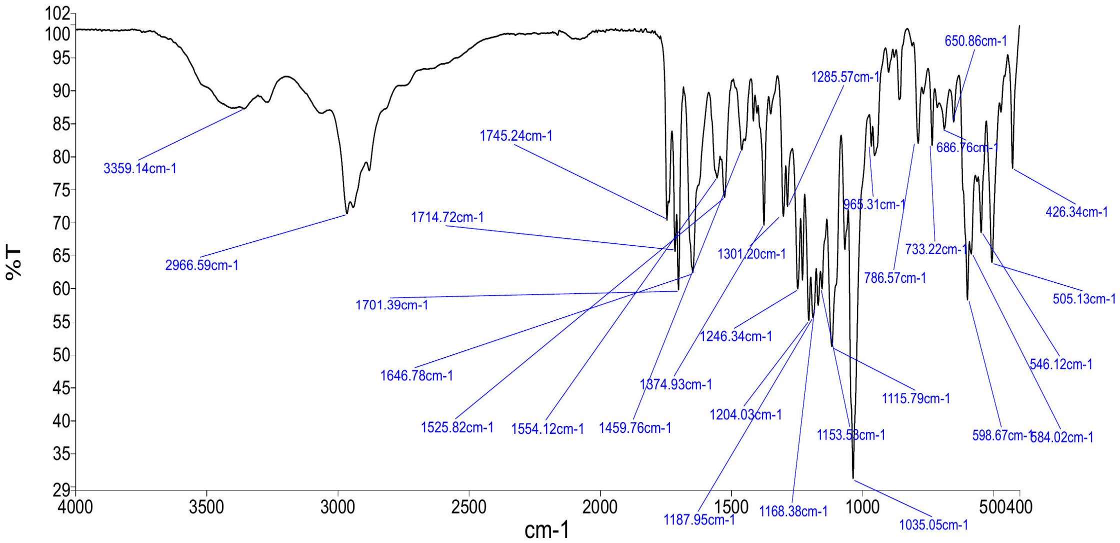

Figure S6. FTIR-ATR spectrum of [OS][CSA].

NMR spectra

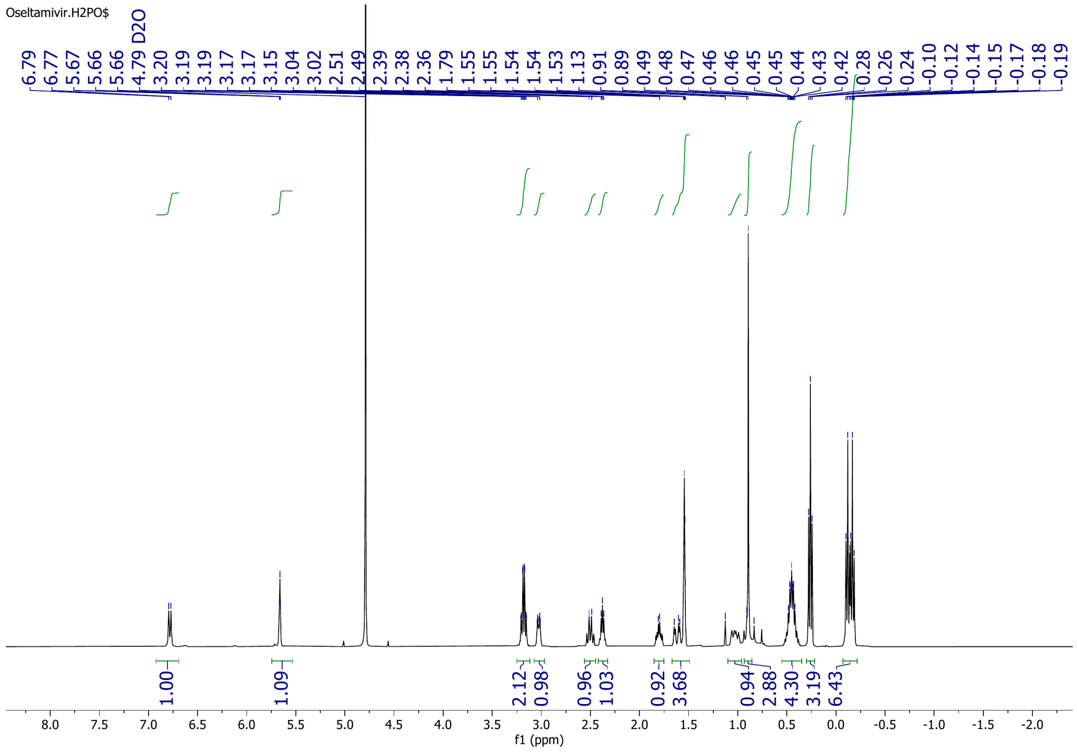

Figure S7. ^1H NMR spectrum of [OS][H_2PO_4] in D_2O.

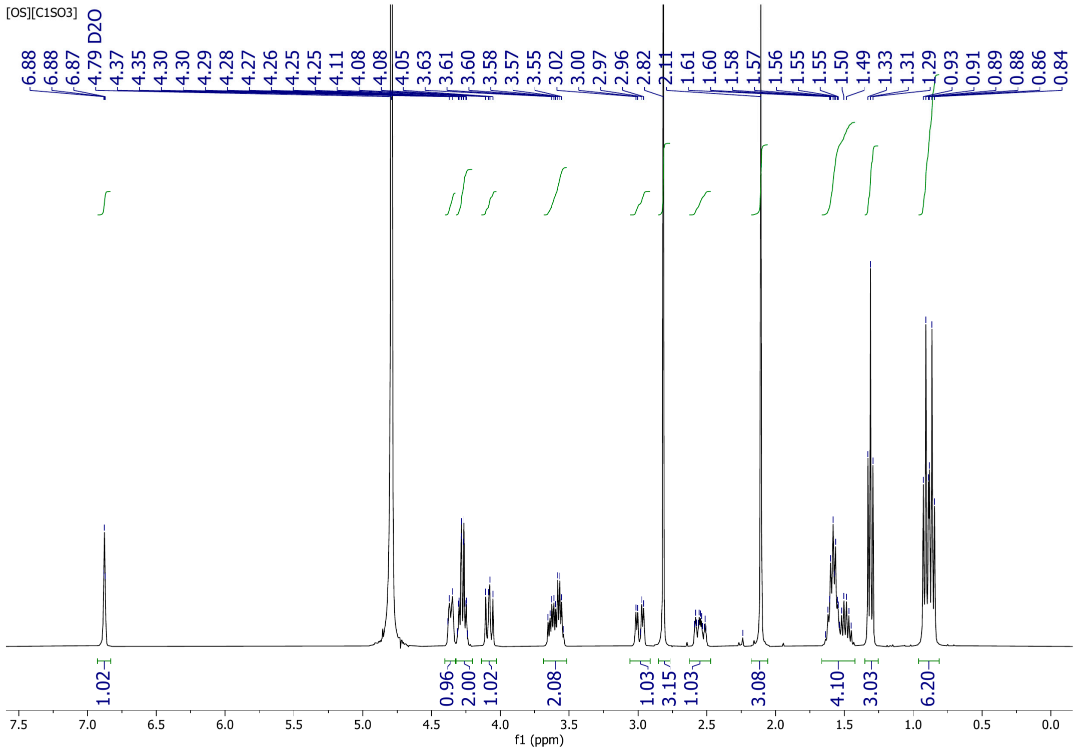

Figure S8. ^1H NMR spectrum of [OS][C_1SO_3] in D_2O.

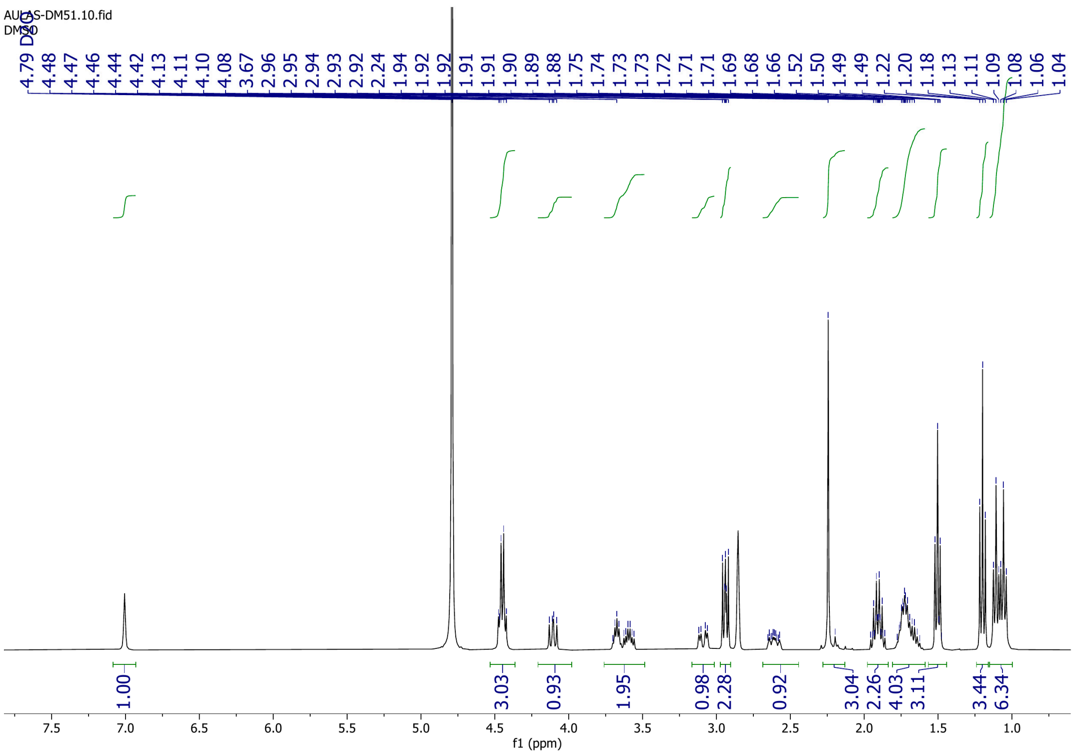

Figure S9. ^{1}H NMR spectrum of [OS][C_3SO_3] in D_2O.

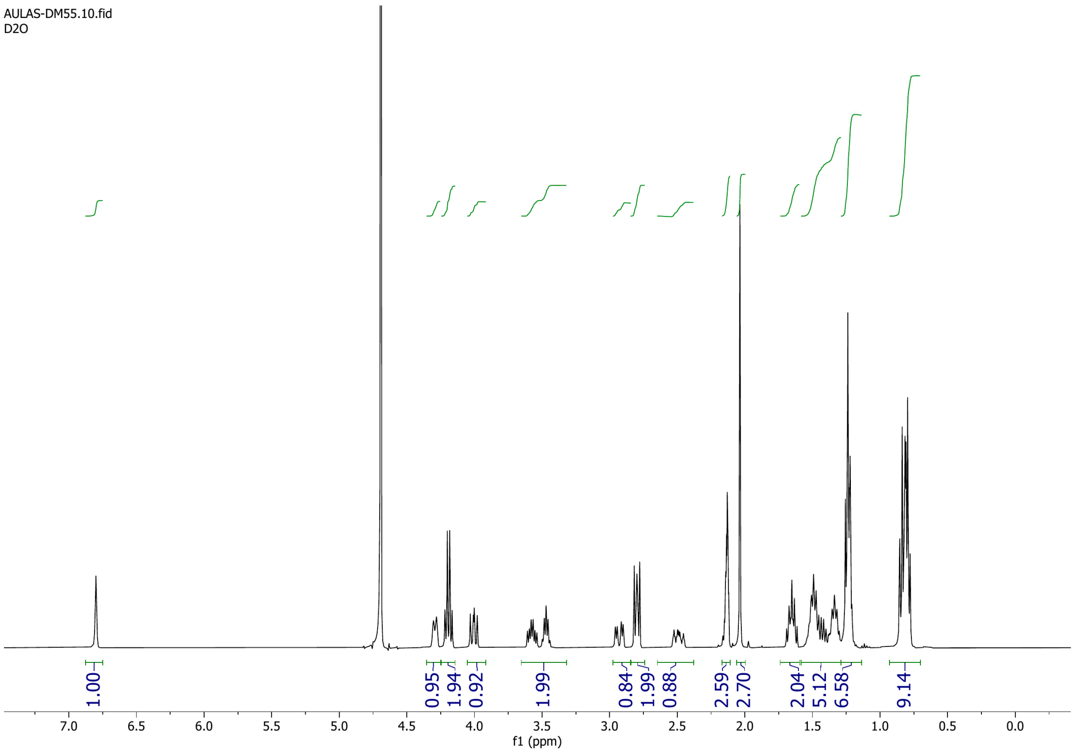

Figure S10. ^{1}H NMR spectrum of [OS][C_6SO_3] in D_2O.

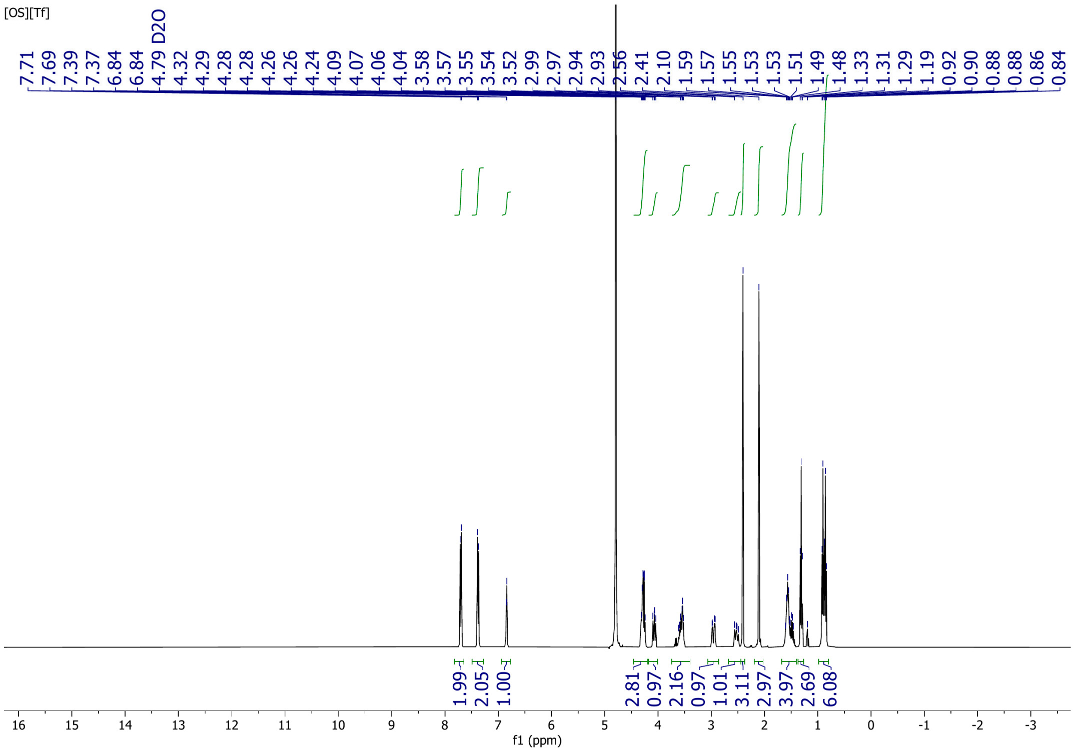

Figure S11. ^{1}H NMR spectrum of [OS][TolSO$_3$] in D_2O.

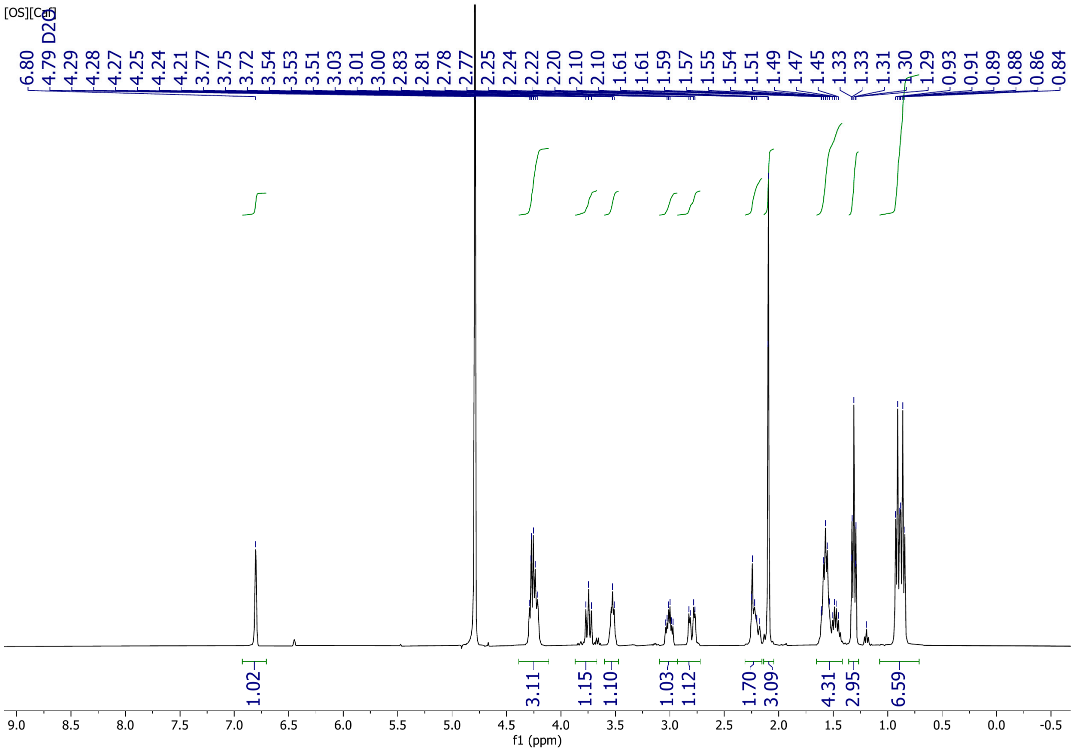

Figure S12. ^{1}H NMR spectrum of [OS][CSA] in D_2O.

^{13}C APT NMR spectra

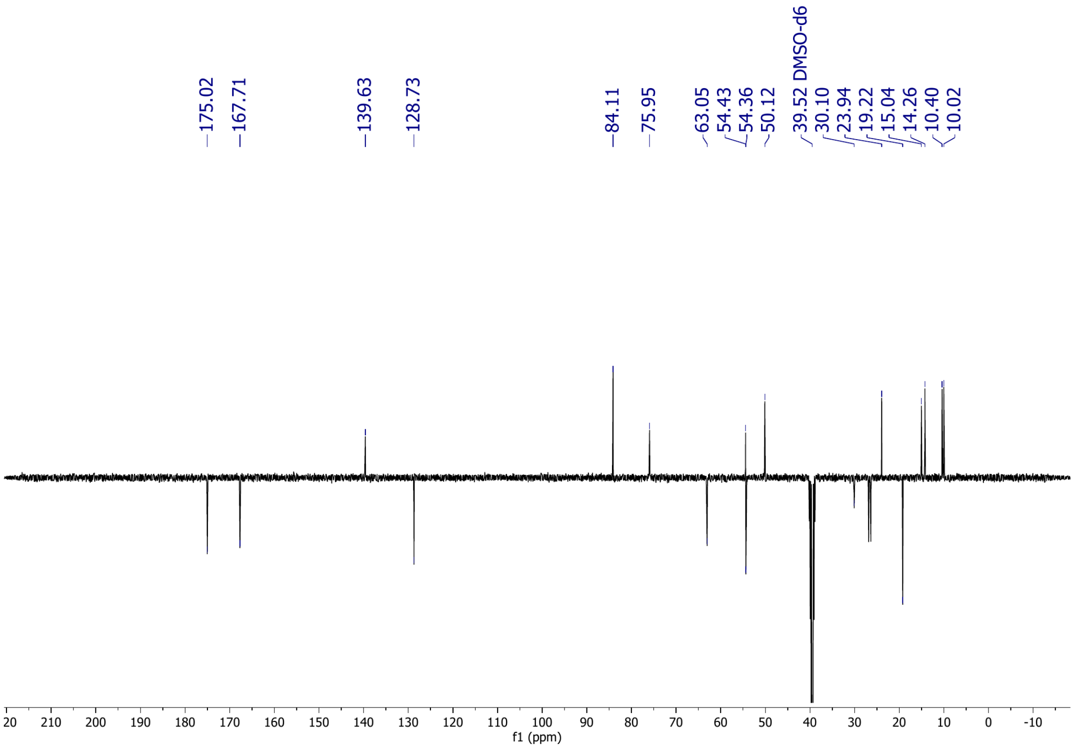

Figure S13. ^{13}C APT NMR spectrum of [OS][H_2PO_4] in D_2O (DMSO-d_6 reference).

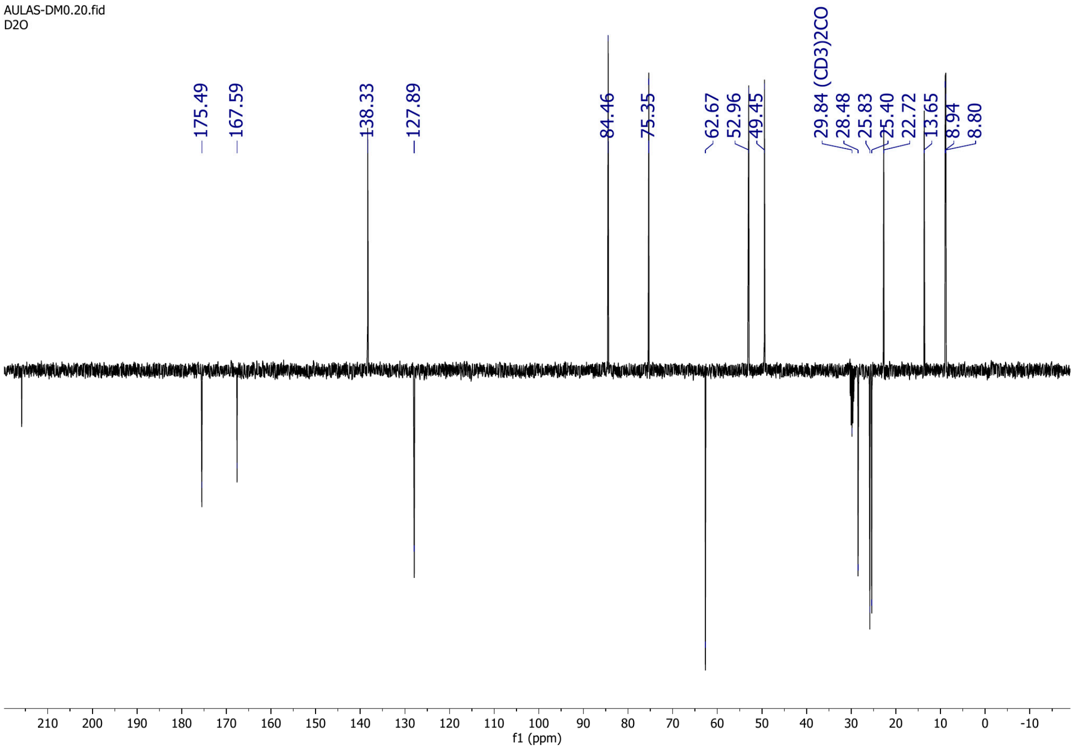

Figure S14. ^{13}C APT NMR spectrum of [OS][C_1SO_3] in D_2O (acetone-d_6 reference).

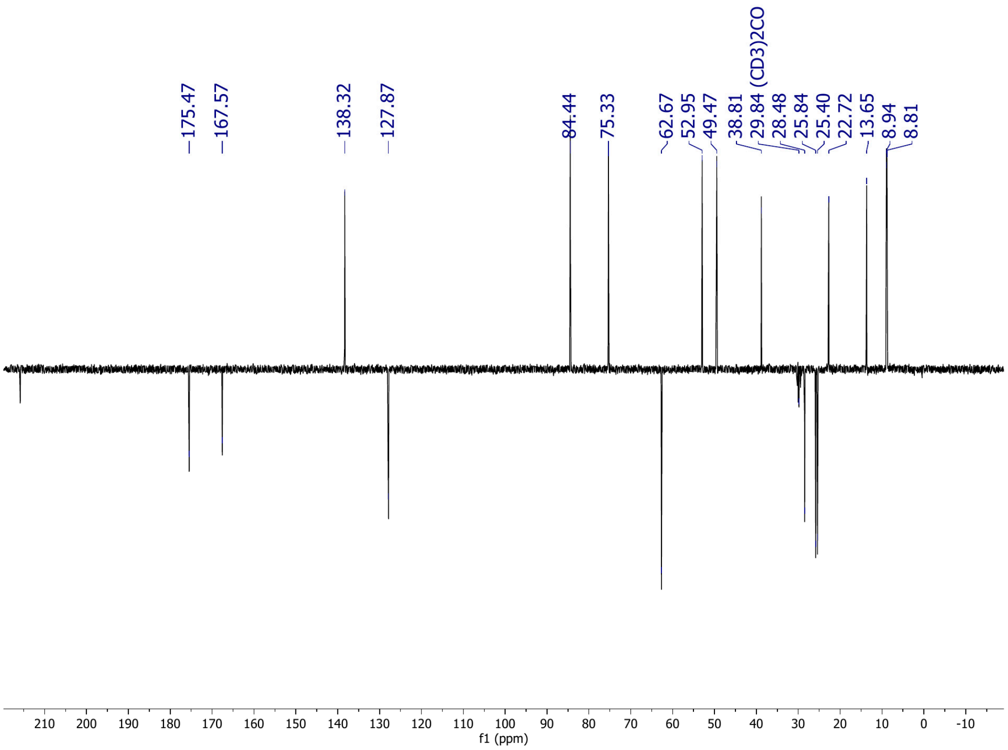

Figure S15. ^{13}C APT NMR spectrum of [OS][C_3SO_3] in D_2O (acetone-d_6 reference).

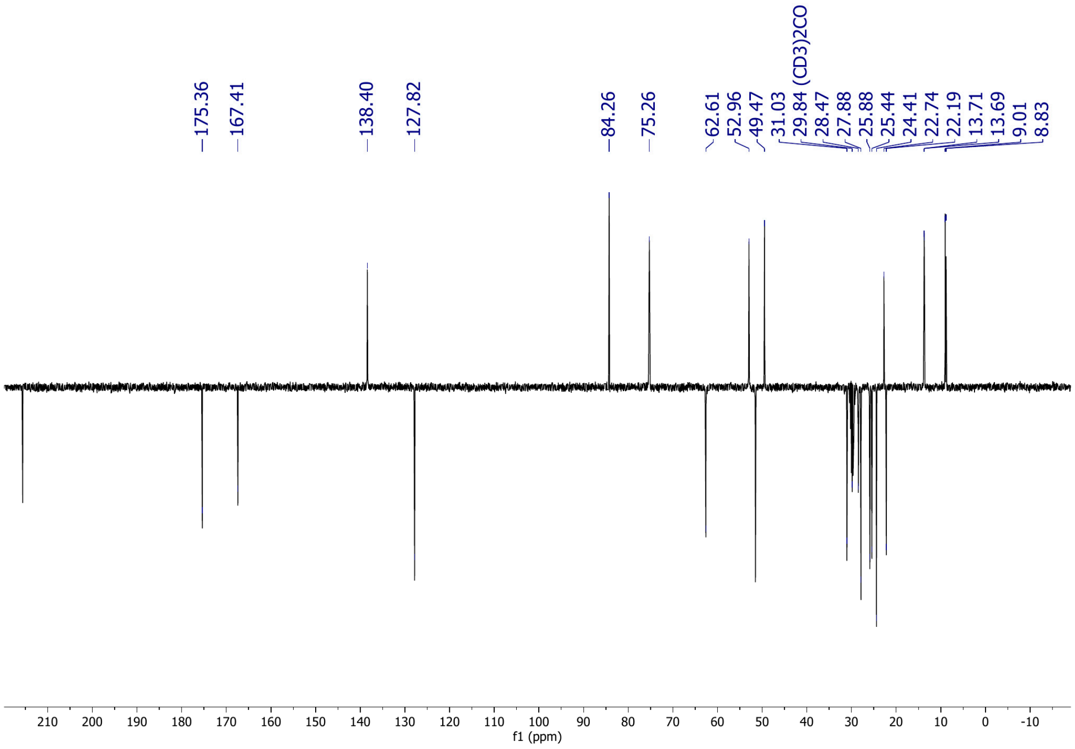

Figure S16. ^{13}C APT NMR spectrum of [OS][C_6SO_3] in D_2O (acetone-d_6 reference).

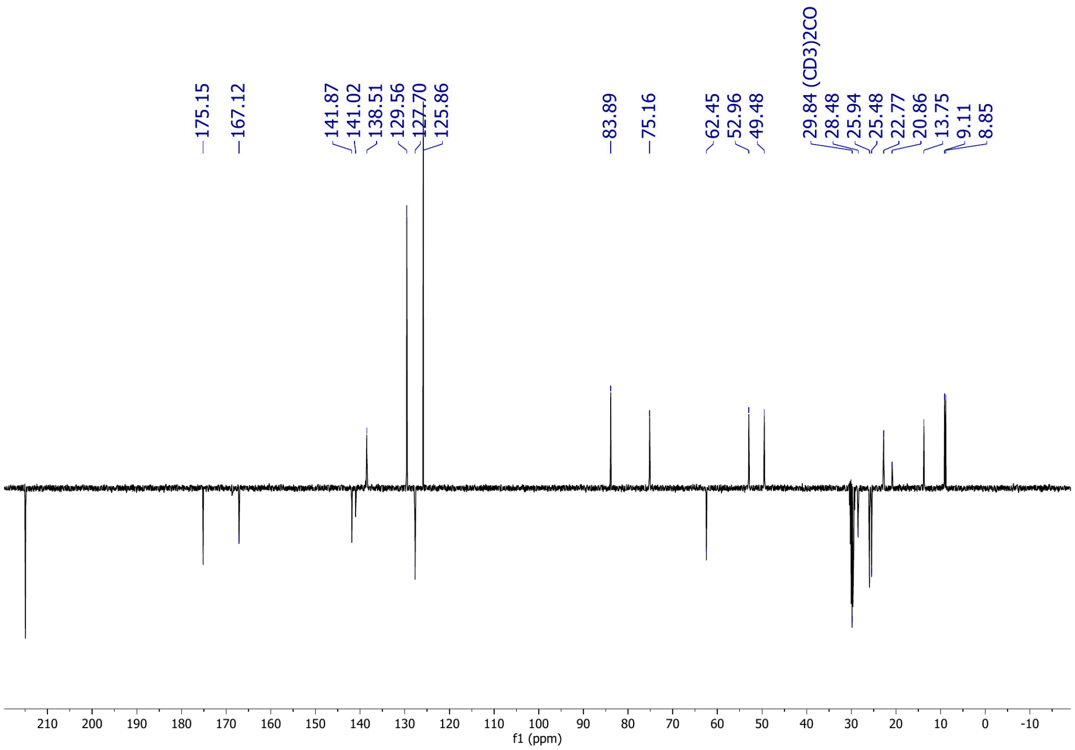

Figure S17. ^{13}C APT NMR spectrum of [OS][TolSO$_3$] in D_2O (acetone-d$_6$ reference).

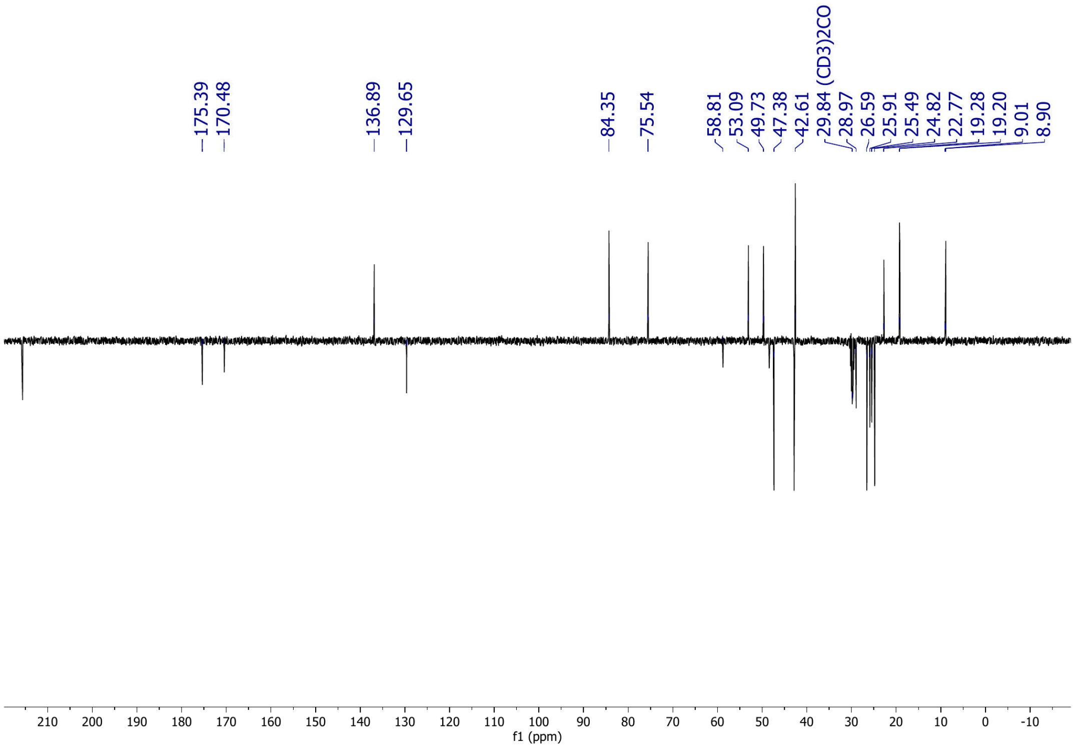

Figure S18. ^{13}C APT NMR spectrum of [OS][CSA] in D_2O (acetone-d$_6$ reference).

Cytotoxicity studies

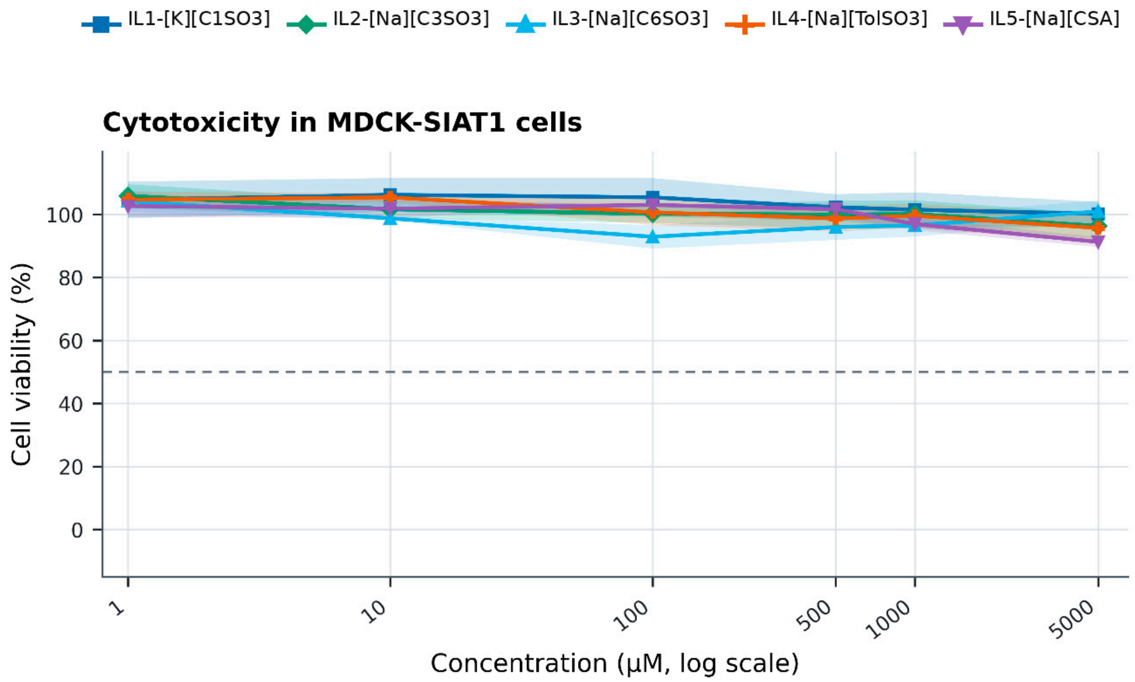

Figure S19. Cytotoxicity of the starting counterions (IL1: [K][C1SO3]; IL2: [Na][C3SO3]; IL3: [Na][C6SO3]; IL4: [Na][TolSO3]; IL5: [Na][CSA]) in MDCK-SIAT1 cells.

Octanol-water partition spectra

The UV-Vis spectra recorded before and after octanol-water partitioning are shown in Figures S20-S25. Abi and ABf represent the initial and final absorbance (at λ_{max}=217 nm) spectra in the water phase, respectively. Each curve is the arithmetic mean of three independent experiments.

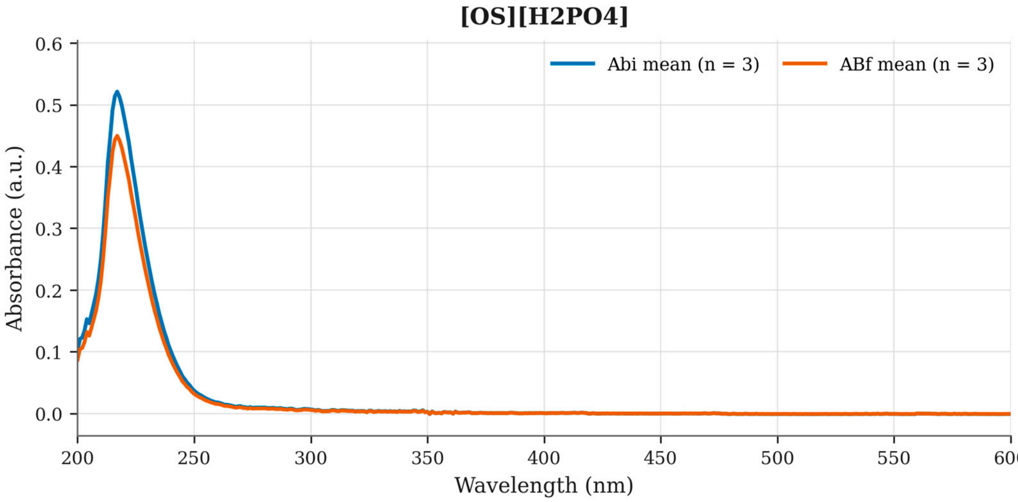

Figure S20. UV-Vis absorbance spectra recorded before (Abi) and after (Abf) octanol-water partitioning for [OS][H2PO4].

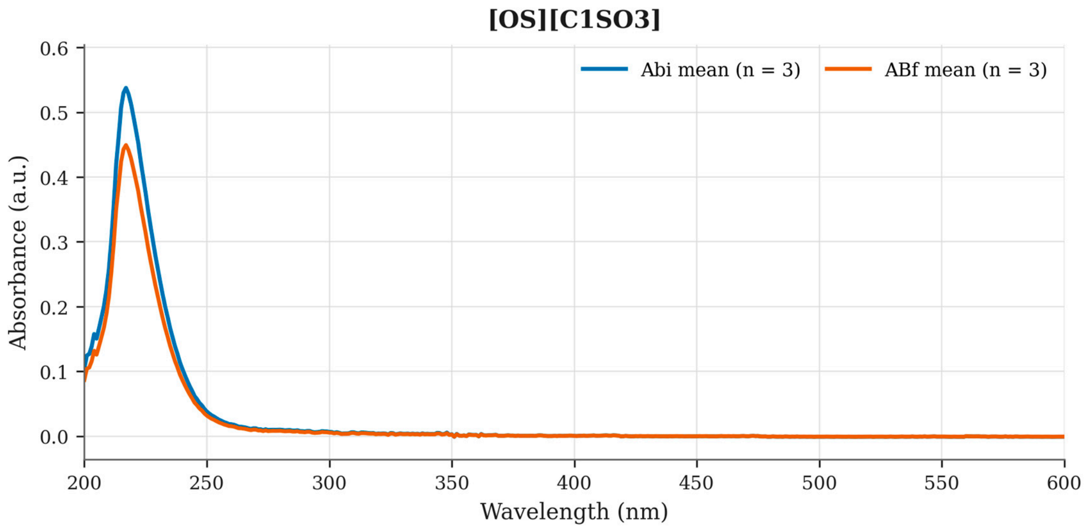

Figure S21. UV-Vis absorbance spectra recorded before (Abi) and after (Abf) octanol-water partitioning for [OS][C1SO3].

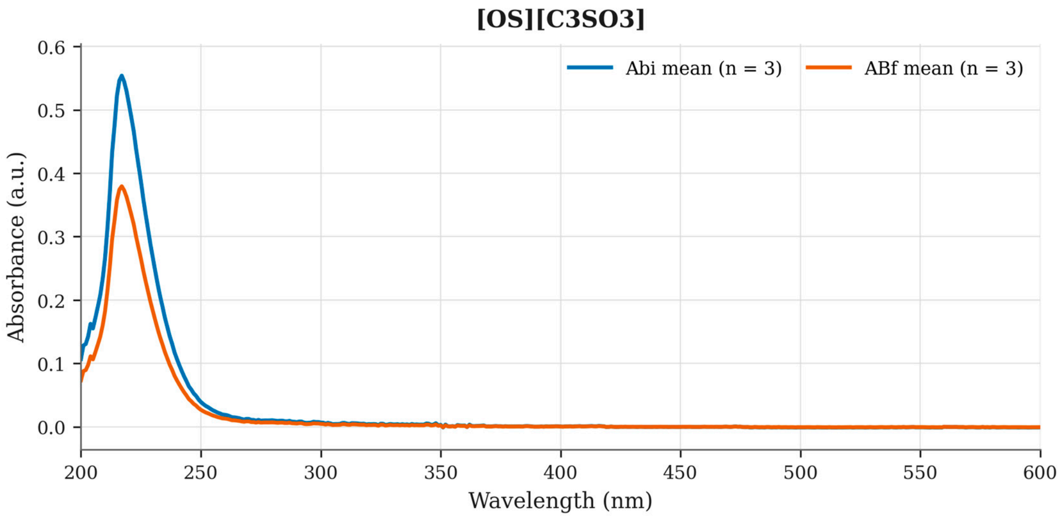

Figure S22. UV-Vis absorbance spectra recorded before (Abi) and after (Abf) octanol-water partitioning for [OS][C3SO3].

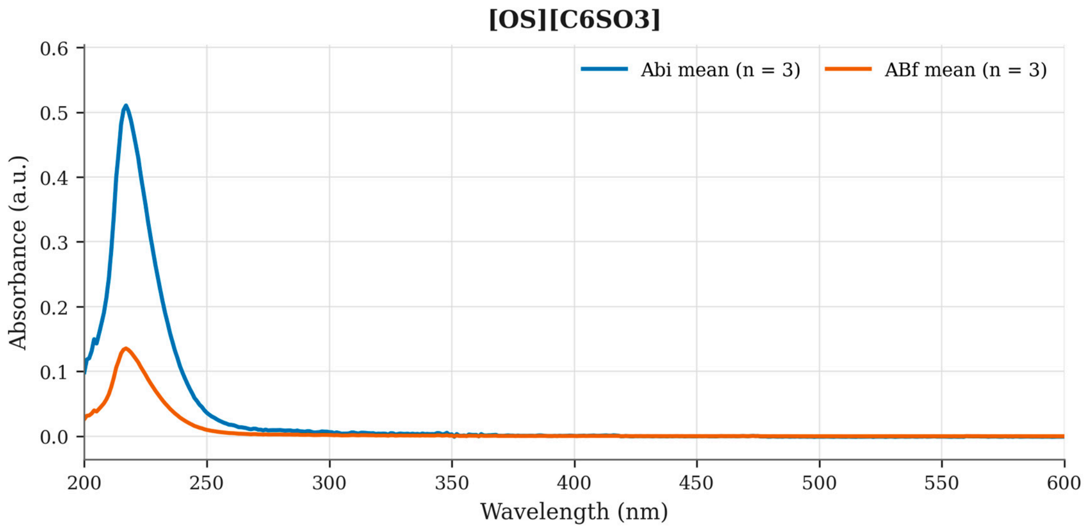

Figure S23. UV-Vis absorbance spectra recorded before (Abi) and after (Abf) octanol-water partitioning for [OS][C6SO3].

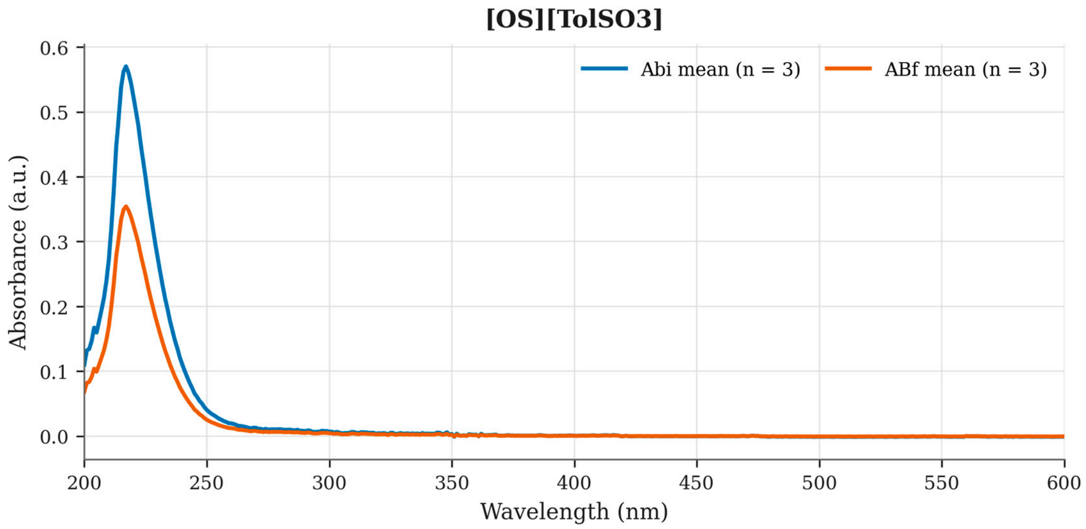

Figure S24. UV-Vis absorbance spectra recorded before (Abi) and after (Abf) octanol-water partitioning for [OS][TolSO3].

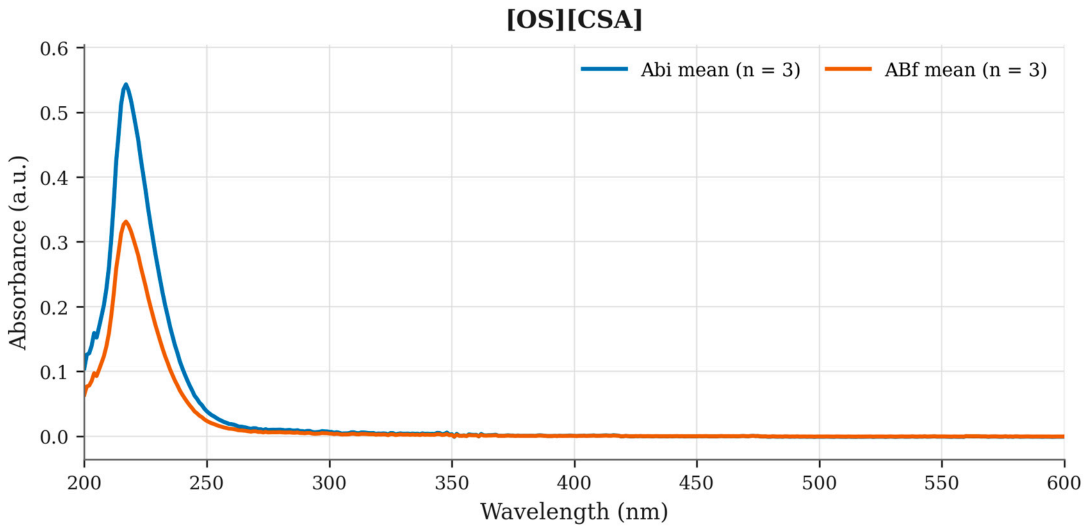

Figure S25. UV-Vis absorbance spectra recorded before (Abi) and after (Abf) octanol-water partitioning for [OS][CSA].
